# Variation in management of febrile infants younger than 90 days across London: a retrospective cohort study

**DOI:** 10.1007/s00431-026-06938-y

**Published:** 2026-05-08

**Authors:** Stephanie Habermann, Rose Hartzenberg, Eva M. Loucaides, George Lawson, Dominic Carr, Ian Maconochie, Ruud G. Nijman, The London REACH Network Collaborative, Nadia Abdulla, Gbemiga Adeoye, Sarah Al Rawi, Mohammad Alam, Sandy Alatzoglou, Humera Ali, Andres Almario, Alexia Alvarez, Alexandros Argyropoulos, Demetris Athanasiou, Nadia Audhali, Nadja Bednarczuk, Shirin Beebeejaun, Sarah Brown, Marnie Bruce, Sarah Chambers, Sabina Checketts, Rhea Clubb, Rosie Crane, Sally-Ann Delaney, Ioannides Demetris, Rhys Dore, Michelle D’Souza, Omar Elsawi, Aderonke Evbuomwan, Sara Farhat Dominguez, Mehreen Farooqi, Margherita Faulkner, Elizabeth Fitchett, Suchika Garg, Neelakshi Ghosh, Claire Girdler, Nuha Gurafi, Eiman Hajahmed, Sarah Hallett, Liza Hallinan, Basma Haroun, Erin Hart, Noha Heikal, Christine Hesketh, Amy Holman, Sasha Howard, Demetris Ioannides, Hannah Jacob, Nasreen Kamarus, Misbah Khan, Sadaf Kiani, Krizstina Kiss, Neave Kissane, May Ko, Louiza Kontara, Gregory Landon, Daniel Langer, Cheryl Lau, Robert Legg, Cheryl Lim, Zhia Lim, Naomi Lin, Conan Lundy, Ashwin Mahtani, Michelle Maria, Jennifer Martin, Kate Mcmonnies, Ramlah Mehmood, Valerie Milton, Basma Mohamed, Abdifatah Mohamud, Claire Mulvenaa, Evangelia Myttarakis, Georgina Nukwe, Fran Neale, Emine Nebati, Maggie Nyirendra, Elizabeth O’Mahoney, Jo O’Sullivan, Ashley Ong, Burcu Ozen, Nana Palm, Konstantina Papagianni, Soonie Patel, Aikaterina Perogiannaki, Caroline Philips, Vasundhara Piyadarshani, Ajith Prasad, Alexia Prol Alvarez, Raj Radhakirshnan, Trisha Radia, Yash Rajeev, Saiqua Raoof, Nilanjana Ray, Jessica Roberts, Hamish Robertson, Jennifer Rossiter, Niamh Scally, Isabel Schulz, Elinor Sefi, Upeka Senanayake, Chid Sethuraman, Jessica Severe, Mashal Shamsuddin, Lucy Shimwell, Khallad Shoaib, Banuja Srikumar, Kokul Sriskandarajah, Anna Starling, Claire Stewart, Claire Strauss, Sarah Sturrock, Constantinos Televantos, Radhika Thakrar, Michelle Themalil, Kyra Theron, Rachel Thompson, Hanna Tilly, Rebecca Unsworth, Kin Wai Man, Marie White, Abigail Whitehouse, Emma Williams, Georgina Yan, Su Ywel, Chris Zhang

**Affiliations:** 1 The London Research, Evaluation and Audit for Child Health (REACH) Network, London, UK; 2 Department of Paediatric Emergency Medicine, Division of Medicine, St. Mary’s Hospital - Imperial College NHS Healthcare Trust, London, UK; 3 Faculty of Medicine, Department of Infectious Diseases, Section of Paediatric Infectious Diseases, Imperial College London, London, UK

**Keywords:** Child health, Emergency care, Paediatrics

## Abstract

Evaluate variation in care of febrile infants presenting to London hospitals and adherence to national Clinical Practice Guidelines (CPGs). Retrospective multicentre cohort study conducted at 21 London hospitals. Infants ≤ 90 days of age presenting with fever or caregiver reported fever between 1st April 2021—31st March 2022 were included. Outcome measures were variations in patient management and adherence to national CPGs. Of 2,008 presentations, 41.1% were febrile during assessment. 73.7% had blood tests (range across hospital sites: 55.4–96.7%), 40.8% had cerebrospinal fluid (CSF) testing (17.1–70.7%), 63.4% had urinalysis (43.4–85.4%) and 62.2% were investigated for SARS-CoV2 (26.5–91.7%). Antibiotics were started in 57.7% (35.4–90.2%) and 63.5% (46.7–99.2%) were admitted. Infants aged < 28 days and infants febrile during assessment were more likely to undergo investigations and be admitted. Adherence varied significantly across sites: full adherence to CPGs was achieved in 21.9% (site range 8.8–35.4%), partial adherence in 24.4% (18.0–35.3%), non-adherence in 31.2% (10.6–47.4%), and over-adherence in 23.5% (8.0–44.7%). CPG adherence was higher in infants aged < 28 days.

*Conclusions*: There was wide variation in care across London hospitals with limited adherence to guidelines. This highlights the need to focus on refining and updating decision models and CPGs, with more specific recommendations for managing infants who are afebrile during initial assessment. Clear guidance with a focus on reducing missed diagnosis may impact patient safety and economical expenditure.

**Supplementary Information:**

The online version contains supplementary material available at 10.1007/s00431-026-06938-y.

**Table:** 

**What is Known:** • *Febrile infants present frequently to emergency departments*. • *10–20% will have a serious bacterial infection (SBI)*. • *Detecting those that need timely investigations, antimicrobial treatment and hospital admission can be challenging*.		
**What is New:** • *This large multicentre study shows wide variation in management across London hospitals*. •* It demonstrates that full adherence to national guidelines is achieved in fewer than a quarter of infants*. • *Adherence is lower in older infants and infants who are febrile at home but afebrile during assessment*.		

## Introduction

Febrile infants present frequently to emergency departments (EDs) [1]. Of those ≤ 90 days old, 10–20% will have a serious bacterial infection (SBI). The majority, however, will have a self-limiting infection [2]. Correctly identifying febrile infants with SBI, who require prompt investigations and antibiotics, is crucial to improving morbidity and mortality. This needs to be balanced against risks of over-investigation and over-treatment.

In the UK, two national Clinical Practice Guidelines (CPGs) support clinicians in the management of febrile infants ≤ 90 days; National Institute for Clinical Excellence (NICE) NG143 (Fever in under 5 s: assessment and initial management) and guidance by the British Society for Antimicrobial Chemotherapy (BSAC) (Guidelines for infants under three months with a fever and no source) [3, 4]. Additionally, some London hospitals use local CPGs, with varied management recommendations [5]. Given the coexistence of multiple national and local guidelines, alongside variable access to diagnostic tests, variation in practice is likely. The extent to which management varies between different hospitals—and whether variation systematically relates to age group, perceived risk or choice of guidelines – remains poorly characterised.

NICE advocates a cautious approach. Following NG143 guidance, 85–100% of febrile infants require parenteral antibiotics despite only 2% having an invasive bacterial infection (IBI) [6, 7]. More tailored approaches include StepByStep, developed in Spain, and the Paediatric Emergency Care Applied Research Network (PECARN), American Association of Pediatrics (AAP) and Aronson CPGs. Following these allows up to 45–54% of infants to be safely discharged without parenteral antibiotics [2, 8–10]. However, many require procalcitonin testing, often not available in UK hospitals.

Internationally, variation in the management of febrile infants has been well described. Studies from North America and Europe have demonstrated significant inter-hospital differences in lumbar puncture rates, antibiotic prescribing, and admission practices, even where national guidelines exist [11–13]. Identified drivers of variation include changing epidemiology [14], poor performance of screening biomarkers [13] and the availability of rapid viral testing [13, 15]. Other variables that have been shown to impact clinical decision making and variation include age, physician’s belief in the validity of the reported fever and physician’s clinical experience [16].

Data describing the extent and drivers of variation within the UK healthcare system remain limited. The aim of the Febrile Infants Regional Evaluation (FIRE) study was to assess variation in the management of infants ≤ 90 days presenting with fever to London hospitals and describe adherence to CPGs. We hypothesised a priori that substantial variation would exist in key management decisions, including investigation strategies, use of parenteral antibiotics, and hospital admission—particularly among infants classified as lower risk. By characterising contemporary UK practice across multiple centres, this study seeks to identify areas of unwarranted variation and inform future efforts to standardise care while maintaining patient safety.

## Methods

### Study design

This retrospective multicentre cohort study was conceived and coordinated by the London Research Evaluation and Audit for Child Health (REACH) network [17]. All London hospitals with a paediatric ED were invited to participate in this study (Supplementary Appendix 1).

### Participants

Infants ≤ 90 days presenting with fever to EDs and acute assessment units from 1 st April 2021 to 31 st March 2022 were identified. Fever was defined as a measured temperature of ≥ 38.0℃ at presentation or as a caregiver-reported temperature of ≥ 38.0℃ in the 12 h preceding presentation, irrespective of method used to measure temperature. Infants who were reported to ‘feel hot’ by their caregiver were included, recognising the importance of parental perception of fever, as per NICE guidelines [3]. Infants born at < 32 weeks gestation and those presenting to neonatal or postnatal wards were excluded.

### Data collection

Collected data included demographics, such as ethnicity and index of multiple deprivations (IMD), and factors influencing susceptibility to infection, for example prematurity, presence of comorbidities, neonatal risk factors for sepsis or vaccination status; and whether infants had received immunisations within 24 h or antipyretics within 8 h before presentation [18–20]. Time of presentation was recorded, differentiating between day (08:00–19:59) and night (20:00–07:59). Data collected included signs and symptoms at presentation, investigations performed (specimen collection and imaging) and management steps, including administration of oral or parenteral antibiotics and admission decision (Supplementary Appendix 2). Outcome data was also collected, including SBI rate – defined as a positive microbiology from an otherwise sterile site and consensus diagnosis – and IBI rate defined as a bacterial meningitis or bacteraemia (non-contaminant) confirmed by culture or molecular diagnostic testing of a sterile site.

Pseudonymised data were extracted from routinely collected clinical records and recorded using a standardised Research Electronic Data Capture (REDCap) form. No patient-identifiable data were stored after pseudonymisation. Infants who re-presented to the same hospital, fulfilling the study inclusion criteria were entered as a new case with a linked study ID.

### Data monitoring and data quality

All data collectors attended training on data collection and entry. The study team (SH, RH) monitored data completeness and accuracy of key variables. Inconsistencies were resolved by contacting local teams and data review.

### Outcome measures

Outcomes were variations in care, specifically investigations, management, and adherence to national CPGs.

### Guideline adherence

The key management steps in NICE and BSAC CPGs were extracted by the study team (RH, SH), and the management of individual cases assessed for adherence to these [3, 4]. For NICE adherence, individual cases were classified as ‘full’, ‘partial’, ‘non-’, or ‘over-adherent’, according to four core investigations (FBC, CRP, blood culture, urinalysis, lumbar puncture (LP)), and receipt of parenteral antibiotics (Supplementary Appendix 3).

### Study size

The aim of this study was to collect data for febrile infants over a 1-year period to describe variation in practice. A scoping and feasibility survey sent to all participating hospitals prior to the study estimated 1980 eligible infants.

### Analyses

Descriptive statistics were calculated using Excel and SPSS (Version 29.0.0.0)). Categorical variables were compared using Chi-square and Fisher’s exact tests, continuous variables using T-tests and Mann–Whitney U tests. Predefined subgroup analyses were performed for (a) infants febrile vs afebrile during assessment (those with a recorded temperature of ≥ 38.0 °C at presentation) compared to infants afebrile during assessment (those with caregiver-reported fever (measured or feeling hot to touch) in the preceding 12 h but afebrile < 38.0 °C during initial assessment); (b) infants aged < 28 days compared with ≥ 28 days at presentation and (c) those presenting during the day (08:00–19:59)) compared with at night (20:00–07:59). Binary logistic regression was performed for analysis of variability in CSF sampling and administration of parenteral antibiotics and of predictors for adherence to NICE CG 143. The binary outcome for adherence to NICE was inappropriate management (defined as either non-adherence, partial adherence and over-adherence to NICE CG 143) and appropriate management (defined as full adherence).

### Ethics

This study was performed in line with the principles of the Declaration of Helsinki. The study protocol was approved with a waiver of patient individual informed consent (REC reference nr: 22/PR/1377), with local approval from research governance offices at all study sites. The study was also registered with the Health Research Authority (Rec reference:16/LO/1522). The Study IRAS project ID is 314,641.

## Results

**Fig. 1:**
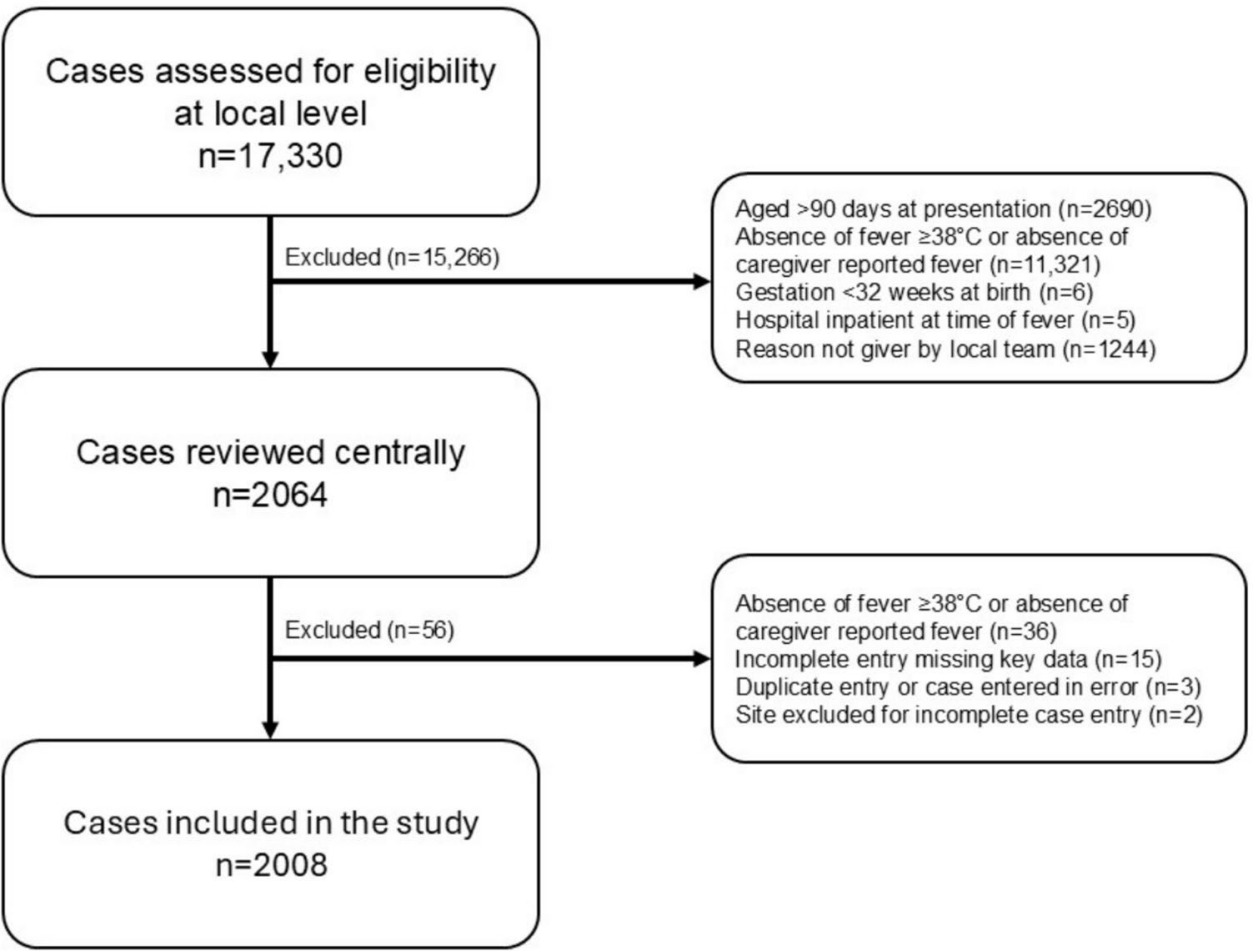
Study flow diagram

17,330 presentations to EDs were identified and assessed for eligibility, 2,008 were included. This comprised 1,900 infants, with 108 infants presenting more than once within the study period (Fig. 1). Of the 108 infants who represented during the study period, only 17 cases (< 1%) represented within 48 h of the first presentation and a total of 42 cases represented within a week of the first presentation (2%).

Table 1 shows cohort characteristics. 826/2,008 (41.1%) were febrile during assessment. Of those with data available, 445/1,372 (32.4%) infants received antipyretics in the 8 h prior to presentation. This was more common in those febrile during assessment than those afebrile (36.0% vs. 27.1%, *p* <.001). The most reported symptoms were reduced feeding (774/2008, 38.5%), coryza (750/2,008, 37.4%) and cough (669/2,008, 33.3%). Fever was the only reported symptom in 262/2,008 (13.0%). On examination 933/2008 (46.5%) infants were well appearing with no localising signs of infection. Additional symptoms, examination findings and vital signs are reported in Table 1 and Supplementary Table S1.

**Table 1:** Cohort characteristics – (**a**) demographic features and (**b**) medical history and presentation features

**a) Demographic features**										
**Whole cohort**		**Age at presentation**			**Fever during assessment** ^a^			**Time of presentation** ^b^		
		** < 28 days**	** ≥ 28 days**		**Febrile**	**Afebrile**		**Day**	**Night**	
*n* = 2,008		*n* = 450	*n* = 1558	*p*	*n* = 826	*n* = 1182	*p*	*n* = 1131	*n* = 877	*p*
Age, median days (IQR)	50 (30–67)	17 (11–23)	58 (45–72)	** <.001**	51 (31–66)	49 (30–67)	.962	48 (28–66)	52 (32–67)	.102
Female, n/N (% total)	811/1986 (40.8%)	174/446 (39.0%)	637/1540 (41.4%)	.374	311/813 (38.3%)	500/1173 (42.6%)	.051	469/1124 (41.7%)	342/826 (39.7%)	.357
Index of multiple deprivation decile, median decile (IQR) ^c^	5 (3–7)	5 (3–7)	5 (3–7)	.503	4 (3–7)	5 (3–7)	**.004**	5 (3–7)	5 (3–7)	.985
Ethnicity ^d^ n/N (% total)										
*White*	903/1756 (51.4%)	197/400 (49.3%)	706/1356 (52.1%)	.544	358/723 (49.5%)	545/1033 52.8%	.558	510/987 51.7%	393/769 (51.1%)	.379
*Asian or Arab*	392/1756 (22.3%)	94/400 (23.5%)	298/1356 (22.0%)		174/723 (24.1%)	218/1033 (21.1%)		229/987 (23.2%)	163/769 (21.2%)	
*Black Caribbean or Black African*	137/1756 (7.8%)	27/400 (6.8%)	110/1356 (8.1%)		55/723 (7.6%)	82/1033 (7.9%)		79/987 (8.0%)	58/769 (7.5%)	
*Other*	175/1756 (10.0%)	42/400 (10.5%)	133/1356 (9.8%)		71/723 (9.8%)	104/1033 (10.1%)		96/987 (9.7%)	79/769 (10.3%)	
*Mixed or Multiple Ethnicities*	149/1756 (8.5%)	40/400 (10.0%)	109/1356 (8.0%)		65/723 (9.0%)	84/1033 (8.1%)		73/987 (7.4%)	76/769 (9.9%)	
**b) Medical history and presenting features**										
**Whole cohort**		**Age at presentation**			**Fever during assessment**			**Time of presentation**		
		** < 28 days**	** ≥ 28 days**	*p*	**Febrile**	**Afebrile**	*p*	**Day**	**Night**	*p*
*n* = 2,008		*n* = 450	*n* = 1558		*n* = 826	*n* = 1182		*n* = 1131	*n* = 877	
Medical history n/N (% total)										
Presence of comorbidities ^e^	304/1941 (15.7%)	40/428 (9.3%)	264/1513 (17.4%)	** <.001**	109/805 (13.5%)	195/1136 (17.2%)	**.030**	181/1092 (16.6%)	123/849 (14.5%)	.209
Preterm ^f^	113/1891 (6.0%)	15/438 (3.4%)	98/1501 (6.5%)	**.015**	46/798 (5.8%)	67/1141 (5.9%)	.921	78/1088 (7.2%)	35/851 (4.1%)	.**004**
Neonatal risk factors for sepsis ^g^	309/1160 (26.6%)	91/336 (27.1%)	218/824 (26.5%)	.827	135/507 (26.6%)	174/653 (**26**.6%)	.994	157/642 (24.5%)	152/518 (29.3%)	.061
Any vaccinations received	580/1664 (34.9%)	11/370 (2.9%)	569/1283 (44.3%)	** <.001**	357/973 (36.7%)	223/691 (32.3%)	.062	282/941 (30.0%)	298/723 (41.2%)	** <.001**
Presenting features n/N (% total)										
Appearing unwell to caregiver	186/2008 (9.3%)	51/450 (11.3%)	135/1558 (8.7%)	.085	88/738 (10.7%)	98/1182 (8.3%)	.072	99/1131 (8.8%)	87/877 (9.9%)	.371
Appears ill to healthcare professional	86/2008 (4.3%)	21/450 (4.7%)	65/1558 (4.2%)	.648	55/826 (6.7%)	31/1182 (2.6%)	** <.001**	50/1131 (4.4%)	36/877 (4.1%)	.741
Initial HR > 160	988/1954 (50.6%)	203/439 (46.2%)	785/1515 (51.8%)	.**045**	585/808 (72.4%)	403/1146 (35.2%)	** <.001**	523/1098 (47.6%)	465/856 (54.3%)	.**003**
Initial RR > 60	126/1938 (6.5%)	41/434 (9.4%)	85/1504 (5.7%)	**.008**	78/805 (9.7%)	48/1133 (4.2%)	** <.001**	57/1090 (5.2%)	69/848 (8.1%)	**.012**

*HR* – heart rate (in beats per minute), *RR* – respiratory rate (in breaths per minute). Categorical variables were compared using Chi-square and Fisher’s exact tests, continuous variables were compared using T-tests and Mann-Whitney U tests, taking into account whether the data were normally distributed

^a^ Fever during assessment was defined as recorded temperature ≥38°C at presentation or at any point whilst in the place of initial assessment (Emergency Department or acute assessment unit). Afebrile at assessment was defined as having a caregiver reported fever or feeling hot to touch in the 12 hours prior to presentation, but temperature <38°C throughout stay in place of initial assessmen

^b^ Day presentation was defined as presenting between 08:00-19:59 and night presentation as presenting between 20:00-07:59

^c^ IMD - index of multiple deprivations (IMD) [20]

^d^ Ethnicity was based on the Office of National Statistics Census 2021 categories and grouped into broader categories for analysis [19]

^e^ A comorbidity was defined as pre-existing conditions, including those present at presentation and those previously present and resolved by the time of presentation

^f^ Preterm was defined as gestation at birth 32+0-36+6 weeks; infants born at <32+0 were excluded from the study

^g^ Neonatal risk factors for sepsis included any risk factor listed in NICE NG195 Neonatal infection: antibiotics for prevention and treatment guideline [21]

### Investigations and management in the whole cohort

**Fig. 2:**
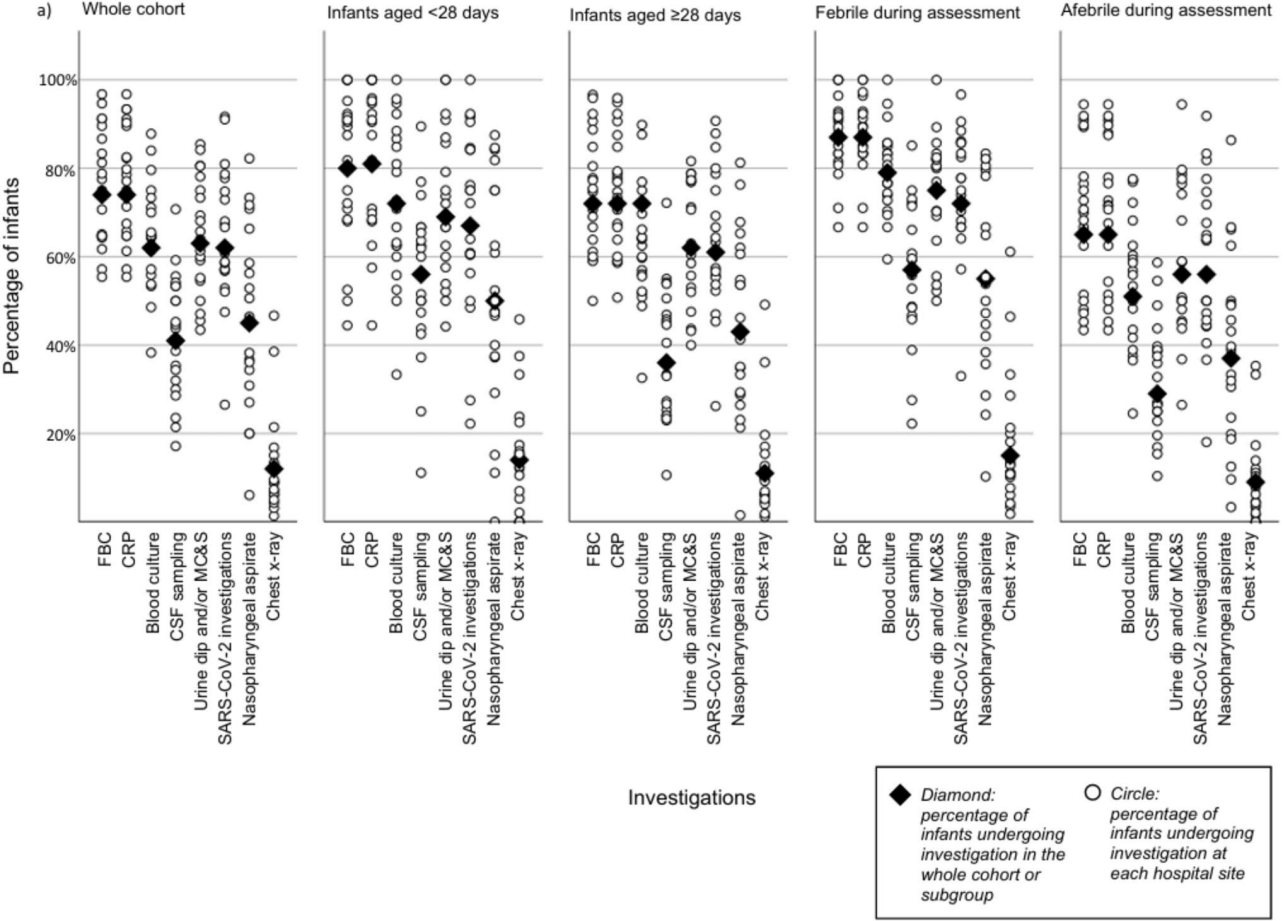

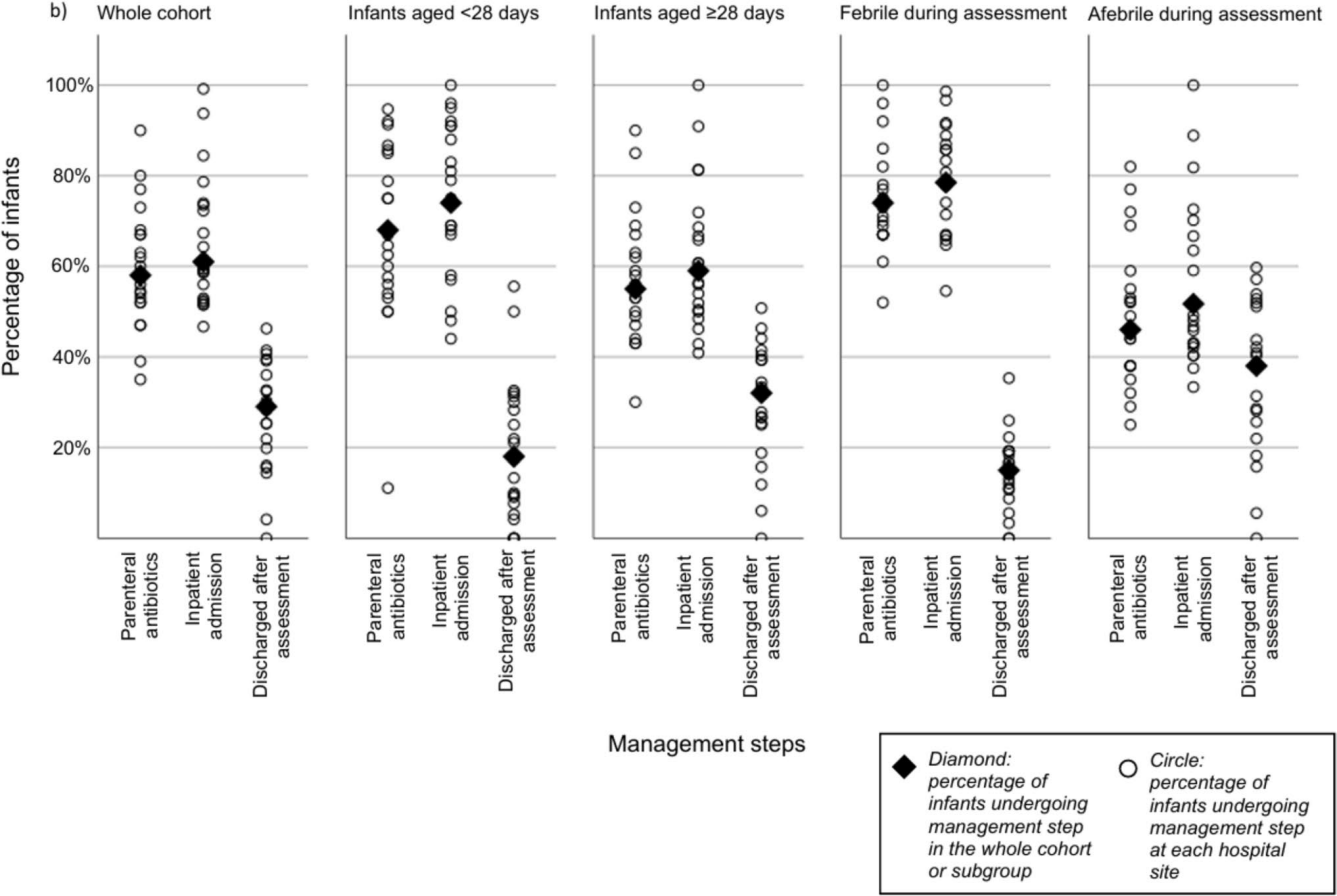
Dot plot showing site-level variation in percentage of infants undergoing (**a**) investigations and (**b**) specific management steps by age category and fever category

FBC and CRP were performed in 1,479/2,008 (73.7%) (range across sites 55.4–96.7%) and 1488/2008 (74.1%) (range 55.4–96.7%), respectively. Blood culture was performed in 1,252/2,008 (62.4%) (range 38.3–87.8%), CSF sampling in 819/2,008 (40.8%) (range 17.1–70.7%), urinalysis in 1,273/2,008 (63.4%) (range 43.4–85.4%), nasopharyngeal aspirate or throat swab in 895/2,008 (44.6%) (range 6.1–82.2%) and SARS-CoV2 investigations in 1,249/2,008 (62.2%) (range 26.5–91.7%). A chest x-ray was performed for 233/2,008 (11.6%) of infants (range 1.4–46.7%), and 85/2,008 (4.2%) had other imaging (Fig. 2A). Of the whole cohort, 1,158/2,008 (57.7%) (range 35.4–90.2%) infants received parenteral antibiotics (Fig. 2B). Most prescribed were cephalosporins (1134/1158, 96.0%), amoxicillin (510/1158, 44.0%) and gentamicin (84/1158, 7.1%). Of those who received parenteral inpatient antibiotics, the median duration of antibiotic administration was 3 days (IQR 2–4), with 9.5% receiving antibiotics for less than 2 days and the top decile receiving antibiotics for > = 5 days. 63.5% (range 46.7–99.2%) of infants were admitted to an inpatient setting, with a median admission duration of 3 days (IQR 1–4), and 1084/1960 (55.3%) admitted for > 24 h. The rate of SBI was 173/2008 (8.6%) and the rate of IBI was 44/2008 (2.2%).

A binary logistic regression analysis of variability in CSF sampling and administration of parenteral antibiotics was performed. (Supplementary Table S2). In this model 19.3% of the variability in the percentage of infants undergoing CSF sampling was explained by the predictors and 17.8% of the variability in the percentage of infants receiving parenteral antibiotics. Significant predictors were age and being febrile at presentation. Hospital sites were included as a predictor in this model and this variable showed overall significance for both CSF sampling and administration of parenteral antibiotics (< 0.001), however in pairwise comparison only one site showed significantly reduced odds of adherence (site I: CSF OR 0.17, CI 0.05–0.59, p 0.005; parenteral antibiotics OR 0.24, CI 0.06–0.96, p 0.029).

### Subgroup analyses

Subgroup analyses by age, fever during assessment, and time of presentation showed similar ethnicity, gender and age distributions. IMD decile differed significantly between those who were febrile during assessment and those who were afebrile (p = 0.004) but was otherwise comparable between subgroups (Table 1). The percentage of infants undergoing FBC, CRP, blood culture, urinalysis and CSF sampling were significantly higher in infants aged < 28 days (Supplementary Table S3)). Infants who were febrile during assessment also underwent significantly more investigations compared to those who were afebrile during assessment (Supplementary Table S3). No significant differences were found in the investigations being undertaken in infants presenting during the day or at night (Supplementary Table S3).

Inpatient admission was significantly higher in those aged < 28 days compared to those ≥ 28 days (74.4% vs. 59.3%, p < 0.001) and those who were febrile during assessment compared to those who were afebrile (78.5% vs 51.7%, p < 0.001). There was no difference in inpatient admission rates between those who presented during the day compared with those who presented at night (62.0% vs. 63.6%, p = 0.45). Higher parenteral antibiotic administration rates were observed in those aged < 28 days (67.8% vs. 54.7%, p < 0.001) and those who were febrile during assessment (73.7% vs. 46.4%, p < 0.001). There was no significant difference between daytime and night-time presentations for parenteral antibiotic prescribing (56.7% vs. 59.0%, p = 0.306) and inpatient admission (62.0% vs. 63.6%, p = 0.45) (Supplementary Table S3).

### Guideline adherence

#### Adherence to specific guideline standards

Adherence to NICE recommended investigation and management steps was highest for FBC (1479/2008, 73.5%), CRP (1488/2008, 74.1%) and administration of parenteral antibiotics (614/833, 73.7%), and lowest in relation to stool culture (37/201, 18.4%) and chest x-ray (40/111, 36.0%). Across all domains, adherence was higher in those who were febrile during assessment. Adherence to parenteral antibiotic administration in infants febrile during assessment was 329/375 (87.7%), and higher in those aged < 28 days (167/182, 91.8%) and those aged ≥ 28 days who appeared unwell (139/167, 92.7%). Adherence to lumbar puncture for any indication was 279/375 (74.4%) in those febrile during assessment, compared to 205/458 (44.8%) in those afebrile during assessment (Table 2A). In those febrile during assessment, adherence to lumbar puncture was highest in those aged < 28 days (146/182, 80.2%), and lowest in those aged ≥ 28 days with an abnormal WCC (115/167, 68.9%).

**Table 2:**
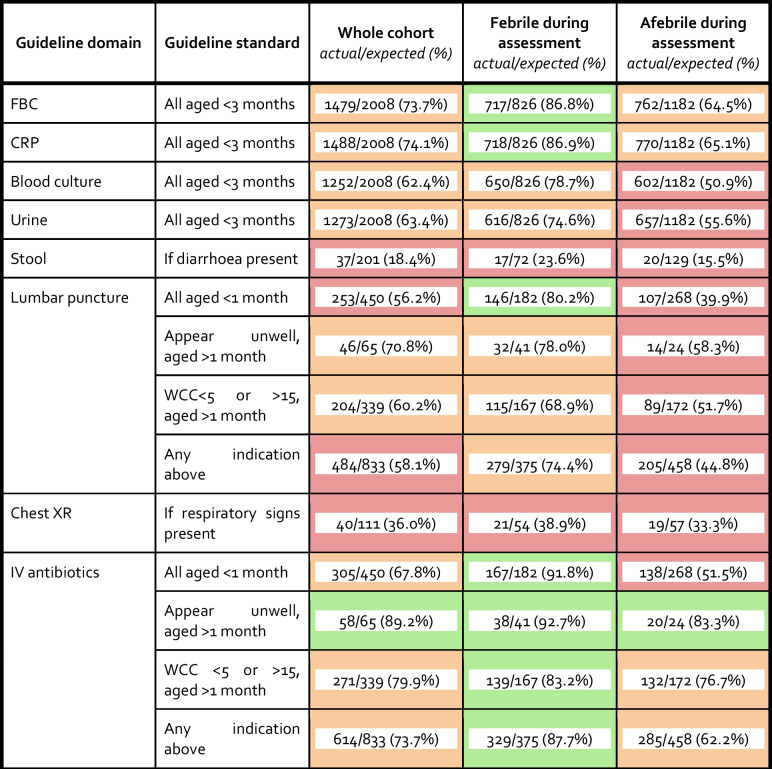
Adherence to national clinical practice guideline NICE CG 143 “Fever in under 5 s: assessment and initial management” in relation to investigation and management of febrile infants

Colour coding: green ≥ 80%, orange 60–80%, red < 60%. FBC full blood count; CRP C-reactive protein; WCC – white cell count; IV intravenous; SBI serious bacterial infection, National Institute for Health and Care Excellence (NICE) NG143 Fever in under 5 s: assessment and initial management [3]

Where the method of temperature measurement was known, only 52% (406/781) of infants afebrile during assessment, had an age-appropriate measurement as per NICE [4].

Guideline standards relating to FBC, CRP, blood culture and urinalysis are the same between NICE and BSAC and were therefore not repeatedly analysed. Adherence rates for LP according to BSAC CPG were similar to NICE adherence rates (Supplementary Table S4). BSAC recommends parenteral antibiotics for moderate and high-risk groups (defined in Supplementary Table S4); higher adherence was observed in those at moderate risk (293/356, 82.3%) compared with those at high risk (398/575, 69.2%).

#### Overall NICE guideline adherence

Full adherence to NG143 was observed in 440/2,008 (21.9%) of infants, with partial adherence accounting for 490/2,008 (24.4%), non-adherence 627/2,008 (31.2%) and over-adherence 451/2,008 (22.5%) of cases (Supplementary Table S5).

Both full adherence (246/826, 29.8%) and over-adherence (239/826, 28.9%) were higher among infants who were febrile during assessment, compared to those who were afebrile (full adherence: 194/1,182, 16.4%; over-adherence: 212/1,182, 17.9%). Non-adherence was less common in those who were febrile during assessment (156/826, 18.9% vs. 471/1,182, 29.8%), and partial adherence was similar (febrile 185/826, 22.4% vs. afebrile 305/1,182, 25.8%). Full adherence was also higher for afebrile infants who had an age appropriate thermometer being used at home (64/292, 21.9%) in comparison to those who were afebrile during the assessment but had an age-inappropriate thermometer used at home (31/299, 10.4%) (Supplementary Table S5 S). For infants afebrile during assessment, who had received antipyretics within 8 h before presentation, full adherence was lower (34/297, 11.4%) compared to those who had not been given antipyretics prior to presenting in hospital (99/529, 18.7%) (Supplementary Table 5).

Full adherence was higher in infants aged < 28 days in comparison to ≥ 28 days (213/450, 47.3% vs. 227/1158, 14.6%; p < 0.001), as was non-adherence (178/450, 39.6% vs. 449/1158, 28.8%; p < 0.001). However, partial adherence was less common (< 28 days 59/450, 13.1% vs. ≥ 28 days 431/1558 27.7%; *p* < 0.001). The highest rate of full adherence was observed in those who were febrile during assessment and aged < 28 days (125/182, 68.7%) (Supplementary Table S5).

When looking at adherence based on NICE decision parameters (lower and higher risk defined in Supplementary Appendix 3), full adherence was markedly higher for the higher risk group (48.4% vs 3.1%) but partial adherence was higher for the lower risk group (29.5% vs 17.2% and 38.4% vs 0%) (Supplementary Table 6).

When adherence was analysed as a binary outcome of appropriately managed (fully adherent) or inappropriately managed (partial, non- and over-adherent) only 37/1175 (3.1%) infants in the lower risk group were managed appropriately in comparison to 403/833 (48.4%) infants in the higher risk group (Supplementary table S6). Binary logistic regression analysis of predictors for adherence to NICE CPG showed that after adjusting for the other variables in the model, being febrile during assessment significantly increased the odds of appropriate management (aOR 2.49, 95% CI 1.92–3.24, *p* < 0.001). Infants aged < 28 days also had significantly increased odds of adherence (OR 5.84, CI 4.45–7.67, p < 0.001). Significantly reduced odds of appropriate management were observed in female gender (aOR 0.73, 95% CI 0.56–0.95, *p* = 0.02) (Supplementary Table S7).

**Fig. 3:**
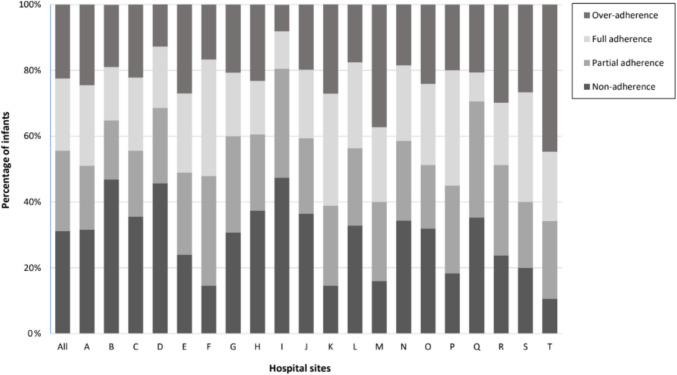
Percentage adherence to the NICE CG 143 guideline across hospital sites

Adherence patterns for the whole cohort varied across sites, with full adherence ranging from 8.8% to 35.4%, partial adherence from 18.0% to 35.3%, non-adherence from 10.6% to 47.4%, and over-adherence from 8.0% to 44.7% (Fig. 3).

## Discussion

This study, looking at young febrile infants in 21 hospitals across London, shows wide variation in care. Mixed adherence to guidelines is demonstrated, with 78% of infants being over- or under-managed. Heterogeneity in management was particularly seen for infants > 28 days old and those afebrile during assessment. This aligns with previous publications, reflecting challenges when managing febrile infants [6, 7, 11–13, 15, 21–23].

There was wide variation in both investigation and management. Some hospitals performed blood tests (FBC and CRP) in 67%of febrile infants, whilst in other hospitals all febrile infants received this test. In our cohort, 63% of infants underwent urinalysis, in contrast with previous studies, in which urine was collected in 81–98% of cases [6, 13]. The significant variation in the percentage of infants undergoing urinalysis (43–85%) was surprising and in contrast to previous reports. All UK CPGs recommend urinalysis in young febrile infants and UTIs comprise 80-90% of SBIs in this age group [12, 13, 24, 25]. Possible explanations for this difference may include challenges in collecting and documenting urinalysis. LP was performed in 41% of infants, similar to another UK study but in contrast to a US study, where 92% infants received LPs [6, 13]. LP rates varied between sites (17–71%), possibly due to clinician attitude. Increasing availability of rapid viral testing, not included in national CPGs, may also impact variation in care [26, 27]. Viral testing was overall common, but rates ranged from 10–80% in our study, similarly to previous findings [13, 28, 29]. Since detecting a viral infection reduces the risk of SBI but does not eliminate it additional guidance is necessary [28–31]. Prescribing practices also varied (37% of infants started on parenteral antibiotics in one hospital, whilst in another hospital, 91% of infants received them). Local variation in practice can result in both under- and over-investigation and management. Under-investigation and treatment may affect patient safety by delaying diagnosis, leading to avoidable complications and prolonged hospital admissions and treatment courses. It may also increase mortality and long-term disability, reduce public trust in healthcare and increase litigation. On the other hand, over-investigation and treatment leads to unnecessary exposure to harm (including complications of procedures, harm from false positives and emotional burden whilst awaiting results), system inefficiency and increased healthcare cost. Additionally, antibiotic use may negatively affect the microbiome and risks contributing to antimicrobial resistance. Infants < 28 days or those febrile during assessment were more likely to undergo investigations, receive antibiotics and be admitted, aligning with previous findings [11, 13, 21]. However, care varied even in these subgroups at higher risk of SBI.

Infants afebrile during assessment (59%of our cohort) underwent significantly fewer investigation and management steps. This aligns with previous studies showing that infants febrile at presentation are five times more likely to have an LP and are significantly more likely to receive antibiotics than those without a fever [32–35]. This is despite NICE recommending that “parental perception of a fever should be considered valid and taken seriously” [3]. Potential reasons include limited evidence about the risk of SBI in those with caregiver reported fever only and potentially inaccurate thermometers [14, 33, 35]. Thirty-six percent of afebrile infants received antipyretics before presentation (of those with available data), suggesting clinicians need to remain vigilant and future recommendations should clarify management for these infants.

Variations may also stem from clinicians' perceptions of risk and their limited experience in managing febrile infants with SBIs. Evidence has shown a decrease in the prevalence of SBI since the introduction of group B streptococcal screening, immunisation against Streptococcus pneumoniae and improvements in food safety [36].

Only twenty-two percent of infants in this study were managed in full adherence to NICE guidelines. Full adherence was further reduced for older infants, afebrile infants, and in particular afebrile infants whose temperature was measured at home with an age-inappropriate thermometer. A potential reason for this is that these infants are perceived to be at a lower risk of SBI by health care professionals. Full adherence was also reduced for those given antipyretics at home. This surprising result could be due to these infants appearing less sick, or maybe due to the fact that they might have had recent immunisations and been advised to give antipyretics.

Adherence to NICE guidance was influenced by age, sex and fever category. Younger infants and febrile infants are more likely to undergo investigations, receive antibiotics and be admitted as previously mentioned which will affect adherence rates; it is unclear why sex affected adherence.

Over-adherence, most common in those febrile at presentation, was most often due to both undertaking a LP and giving parenteral antibiotics. This is clinically harmful. Adverse effects from LP and antibiotics occur and admissions are prolonged. It therefore poses a risk to patient safety and negatively affects resource utilisation. Strategies need to be developed on how to improve patient selection, considering clinical gestalt and new internationally validated risk stratification tools [2, 8–10]. Interestingly, no differences in management or guideline adherence were found between daytime or nighttime presentations. It is uncommon that senior clinicians are present overnight, suggesting clinician experience does not fully explain variation in practice.

Discrepancies between local and national CPGs may contribute to the high variability and low adherence [5, 15, 18, 20–23]. Current UK-wide CPGs have high sensitivity but low specificity, meaning invasive procedures are often performed on infants who do not have an SBI [6]. Clinician gestalt, a synthesis of objective findings and their overall impression, may offer a better sensitivity–specificity balance than CPGs, with similar sensitivity but higher specificity. This could, potentially reducing invasive procedures and antibiotic prescriptions [7]. This may reflect clinicians integrating CPG principles with experience and signs not easily captured by guidelines.

A strength of this study is the large size and complete cohort of consecutive young febrile infants, with data from 21 regional teaching and district general hospitals. This makes the results more generalisable. In contrast, previous studies mostly included teaching or tertiary hospitals [7, 13, 24]. This study also included infants with caregiver reported fever, representing a subgroup with substantial management variability, reflective of real-world experience. The retrospective nature of the study imposes limitations on data quality, particularly with respect to variables that rely on potentially subjective documentation of patient history and examination findings. Another limitation is that data on some hospital variables including ED workload, or clinicians’ seniority level was not collected.

## Conclusion

We describe wide variation in care across London hospitals with inconsistent adherence to guidelines. These findings suggest that current decision models and clinical practice guidelines (CPGs) may benefit from review and updating to reflect contemporary SBI epidemiology, point-of-care viral and newer molecular tests, and more specific advice on the management of infants who are afebrile during assessment in hospital. Identifying factors affecting management decisions and guideline non-adherence in a mixed-methods approach will be important. Updated CPGs, leading to improved adherence, could reduce unnecessary investigation, antibiotic exposure and hospital admission, without increasing the number of missed SBI cases in low-risk, febrile infants [37, 38]. Reducing variation in care may have implications for scarce resources and may contribute to improved and more equitable experiences and outcomes for febrile infants and their families.

## Supplementary Information

Below is the link to the electronic supplementary material.

Supplementary file1 (DOCX 6393 KB)

## Data Availability

All data supporting the findings of this study are available within the paper and its Supplementary Information.

## Abbreviations

AAP: American Association of Pediatrics
BSAC: British Society for Antimicrobial Chemotherapy
CPGs: Clinical Practice Guidelines
CRP: C-reactive protein
CSF: Cerebrospinal-fluid
ED: Emergency Departments
FBC: Full Blood Count
FIRE: Febrile Infants Regional Evaluation
HR: Heart Rate (in beats per minute)
IBI: Invasive Bacterial Infection
IMD: Index of Multiple Deprivations
LP: Lumbar Puncture
MC&S: Microscopy, Culture and Sensitivity
NICE: National Institute for Clinical Excellence
PECARN: Paediatric Emergency Care Applied Research Network
REACH: Research Evaluation and Audit for Child Health
REDCap: Research Electronic Data Capture
RR: Respiratory Rate (in breaths per minute)
SARS: CoV2 severe acute respiratory syndrome coronavirus 2
SBI: Serious Bacterial Infection
UTI: Urinary Tract Infection
WCC: White cell count
