# Supplementary material for: Variation in management of febrile infants younger than 90 days across London: a retrospective cohort study
Source: Eur J Pediatr. 2026 May 8;185(6):370. doi: 10.1007/s00431-026-06938-y (PMC13156226; doi:10.1007/s00431-026-06938-y)
Supplement: Supplementary file 1 — Supplementary file1 (DOCX 6393 KB) [file 431_2026_6938_MOESM1_ESM.docx]

**SUPPLEMENTARY APPENDIX**

**Variation in management of febrile infants younger than 90 days across London: a retrospective cohort study**

Rose Hartzenberg, Stephanie Habermann, Eva Loucaides, George Lawson, Dominic Carr, Ian Maconochie, Ruud Nijman, The London REACH Network Collaborative

Table of Contents

[Supplementary Appendix 1 – Study site recruitment](#_Toc222847066)

[Supplementary Appendix 2 – FIRE study Case Record Form](#_Toc222847067)

[Supplementary Appendix 3 – NICE Guideline CG 143 adherence definitions](#_Toc222847068)

[Supplementary Table S1 – Cohort characteristics: additional presenting features](#_Toc222847069)

[Supplementary Table S2 – Comparison of investigations and management between subgroups](#_Toc222847070)

[Supplementary Table S3 – binary logistic regression analysis of variability in CSF sampling and administration of parenteral antibiotics](#_Toc222847071)

[Supplementary Table S4 – Adherence to national clinical practice guideline BSAC “Guideline for infants under 3 months with a fever and no source” in relation to investigation and management of febrile infants](#_Toc222847072)

[Supplementary Table S5 – NICE Guideline CG 143 adherence by age and fever during assessment](#_Toc222847073)

[Supplementary Table S6 – NICE Guideline CG 143 adherence by risk group](#_Toc222847074)

[Supplementary Table S7 - Binary logistic regression analysis of predictors for adherence (appropriate vs. inappropriate management) to NICE guideline CG 143](#_Toc222847075)

# Supplementary Appendix 1 – Study site recruitment

We invited all London hospitals with a paediatric emergency department to participate in the FIRE study and recruited paediatric resident doctors to volunteer as local leads under the supervision of a paediatric consultant. Local leads then completed a scoping and feasibility survey to establish feasibility of local data collection and to aid study sample size estimation. Of 26 hospitals, 21 were included in the study.

# Supplementary Appendix 2 – FIRE study Case Record Form

## Identifiers

| **#** | **Variable Name** | **Field Label / Field Note** | **Field Attributes / Choices** |
| --- | --- | --- | --- |
| 1 | [id_redcap] | Redcap ID | text |
| 2 | [id_initial] | Initials of team member entering data *Initials of team member responsible for data entry* | text |
| 3 | [id_date] | Date of data entry | text (date_dmy) |
| 4 | [id_studyid] | Study ID *Format XX-YYY-ZZ. XX=site ID, YYY=patient ID, ZZ=hospital attendance (e.g. for an infant who has two eligible presentations within the study period these would be entered separately 01 and 02)* | text |
| 5 | [id_attnumb] | Total number of hospital attendances during the study period *This should be the total number of times the infant has presented as a case attending your hospital between 01/04/2021 to 31/03/2022 i.e. aged <=90 days and febrile (as per study inclusion criteria)* | text (integer, Min: 1, Max: 10) |
| 6 | [id_pres_datetime] | Date and time of presentation | text (datetime_ymd) |
| 7 | [id_pres_loc] | Location of presentation *This is the location where the infant was first seen and underwent assessment by a healthcare professional* | dropdown 1 Emergency department 2 Paediatric assessment unit 3 Paediatric inpatient ward 4 Other |
| 8 | [id_pres_locoth] | If other, please provide further details *Show the field ONLY if: [id_pres_loc] = '4'* | text |
| 9 | [identifiers_complete] | Complete? | dropdown 0 Incomplete 1 Unverified 2 Complete |

## Demographics

| **#** | **Variable Name** | **Field Label / Field Note** | **Field Attributes / Choices** |
| --- | --- | --- | --- |
| 10 | [dem_pres_age] | Age at presentation *This should be entered in days at time of presentation using Age Calculator.* | text (integer, Min: 1, Max: 90) |
| 11 | [dem_sex] | Sex at birth | radio 1 Male 2 Female 3 Data not available |
| 12 | [dem_ethn] | Ethnicity | dropdown 1 White English/Welsh/Scottish/Northern Irish/British 2 White Irish 3 White Gypsy or Irish Traveller 4 Any other White background 5 Mixed/Multiple ethnic groups: White and Black Caribbean 6 White and Black Caribbean: White and Black African 7 White and Black Caribbean: White and Asian 8 Any other Mixed/Multiple ethnic background 9 Asian/Asian British: Indian 10 Asian/Asian British: Pakistani 11 Asian/Asian British: Bangladeshi 12 Asian/Asian British: Chinese 13 Any other Asian background 14 Black/African/Caribbean/Black British: African 15 Black/African/Caribbean/Black British: Caribbean 16 Any other Black/African/Caribbean background 17 Other ethnic group: Arab 18 Any other ethnic group 19 Data not available |
| 13 | [dem_imdd] | Index of multiple deprivation decile *Please use Index of Multiple Deprivation Calculator. Enter postcode into the calculator and record the decile.* | dropdown 1-10 |
| 14 | [demographics_complete] | Complete? | dropdown 0 Incomplete 1 Unverified 2 Complete |

## Birth History

| **#** | **Variable Name** | **Field Label / Field Note** | **Field Attributes / Choices** |
| --- | --- | --- | --- |
| 15 | [bh_birth_gest] | Was the infant born at term (37 weeks or more) or prematurely (32+0 to 36+6 weeks)? *Term includes any infant born at or above 37+0 weeks gestation. Preterm includes any infant born at 32+0 up to and including 36+6 weeks. Please note, infants born before 32 weeks gestation are excluded from the study.* | radio 1 Term 2 Preterm 3 Data not available |
| 16 | [bh_birth_gestweek] | What was the gestation at birth? *Enter in the format ww+d (e.g. 34+5). If days not recorded in documentation enter completed weeks gestation (e.g. 34+0)* | text |
| 17 | [bh_birth_nnu] | Was the infant admitted to the neonatal unit? *Includes any neonatal unit admission including SCBU, HDU and NICU* | radio 1 Yes 2 No 3 Data not available |
| 18 | [bh_birth_abx] | Did the infant receive any antibiotics postnatally? | radio 1 Yes 2 No 3 Data not available |
| 19 | [bh_rffs_any] | Were any risk factors for sepsis documented? | radio 1 Yes 2 No - 'no risk factors for sepsis' documented 3 Data not available |
| 20 | [bh_rffs_which] | Which of the following risk factors for sepsis were documented? *Show the field ONLY if: [bh_rffs_any] = '1'. These questions relate to information that was recorded in the patient's notes relating to this hospital attendance and does not require you to access maternal or neonatal records* | checkbox 1 Suspected or confirmed infection in another baby in the case of a multiple pregnancy 2 Invasive group B streptococcal infection in a previous baby OR maternal group B streptococcal colonisation, bacteriuria or infection in the current pregnancy 3 Pre-term birth following spontaneous labour before 37 weeks gestation 4 Confirmed rupture of membranes for >18h before a pre-term birth 5 Confirmed prelabour rupture of membranes at term for >24h before the onset of labour 6 Intrapartum fever >38 |
| 21 | [bh_rffs_other] | If other, please provide further details *Show the field ONLY if: [bh_rffs_any] = '1'* | text |
| 22 | [birth_history_complete] | Complete? | dropdown 0 Incomplete 1 Unverified 2 Complete |

## Medical History

| **#** | **Variable Name** | **Field Label / Field Note** | **Field Attributes / Choices** |
| --- | --- | --- | --- |
| 23 | [mh_comorb_any] | Did the infant have any other medical conditions recorded at the time of hospital attendance? *Includes any pre-existing conditions that were present at the time of attendance and conditions which were present previously and had resolved by the time of attendance* | radio 1 Yes 2 No 3 Data not available |
| 24 | [mh_comorb_spec] | Please select the relevant medical conditions *Show the field ONLY if: [mh_comorb_any] = '1'. Select all that apply based on information available in the patient's records. Congenital anomaly = present at birth; includes genetic syndromes, inborn errors of metabolism and structural abnormalities. Jaundice = jaundice requiring phototherapy or prolonged jaundice.* | checkbox 1 Gastroesophageal reflux disease 2 Cows milk protein intolerance 3 Eczema 4 Jaundice 5 Colic 6 Congenital abnormalities 7 Other 8 No underlying comorbidities 9 Data not available |
| 25 | [mh_comorb_sys] | Which systems were involved in the infant's other medical conditions? *Show the field ONLY if: [mh_comorb_any] = '1'. Select all that apply* | checkbox 1 Pulmonary 2 Cardiac 3 Gastro/nutrition 4 Liver 5 Surgical 6 Neurologic 7 Metabolic 8 Endocrine 9 Oncological 10 Psychomotor delay 11 Immunodeficiency 12 Haematological 13 Other 14 No underlying comorbidities 15 Data not available |
| 26 | [mh_comorb_det] | Please provide further details *Show the field ONLY if: [mh_comorb_any] = '1'* | text |
| 27 | [mh_meds_any] | Was the infant receiving any regular medications at the time of presentation? *Includes prophylactic antimicrobials* | radio 1 Yes 2 No 3 Data not available |
| 28 | [mh_meds_det] | Please provide further details *Show the field ONLY if: [mh_meds_any] = '1'* | text |
| 29 | [medical_history_complete] | Complete? | dropdown 0 Incomplete 1 Unverified 2 Complete |

## Vaccinations

| **#** | **Variable Name** | **Field Label / Field Note** | **Field Attributes / Choices** |
| --- | --- | --- | --- |
| 30 | [vacc_any] | Had the infant received any vaccinations up to the date of hospital attendance? *This includes any vaccination given from birth to the time of hospital attendance (vitamin K is not included).* | radio 1 Yes 2 No 3 Data not available |
| 31 | [vacc_which] | Which vaccinations had the infant received up to the date of hospital attendance? *Show the field ONLY if: [vacc_any] = '1'. Select all that apply. 'Other' should be selected if the infant has received other vaccinations such as hepatitis B or if they've received incomplete vaccinations (i.e. single doses). 'Immunisations up-to-date' should be selected if clerking notes document 'immunisations up-to-date' without further detail* | checkbox 1 BCG 2 8 week (DTaP/IPV/Hib/HepB, MenB, Rota) 3 12 week (DTaP/IPV/Hib/HepB, PCV, Rotavirus) 4 Other 5 Not applicable 6 'Immunisations up-to-date' 7 Data not available |
| 32 | [vacc_rec] | Were any vaccinations received in the 24 hours prior to presentation? *Show the field ONLY if: [vacc_any] = '1'* | radio 1 Yes 2 No 3 Data not available |
| 33 | [vacc_mat] | Were any of the following vaccinations received during pregnancy? *This should be based only on documentation in the infant's medical notes. Maternal records should not be accessed. If not documented in the infant's notes please select 'Data not available'.* | checkbox 1 Influenza 2 Pertussis 3 Covid-19 4 None 5 Data not available |
| 34 | [vaccinations_complete] | Complete? | dropdown 0 Incomplete 1 Unverified 2 Complete |

## Presenting Features

| **#** | **Variable Name** | **Field Label / Field Note** | **Field Attributes / Choices** |
| --- | --- | --- | --- |
| 35 | [pf_fever_pres] | Was a fever reported by caregivers in the 12 hours preceding presentation? *This includes any recorded temperature of 38.0 degrees or more and any subjective reports of fever (i.e. the child 'felt hot') with or without a measured temperature* | radio 1 Yes 2 No 3 Data not available |
| 36 | [pf_fever_max] | What was the maximum temperature reported by caregiver prior to presentation? *Show the field ONLY if: [pf_fever_pres] = '1'* | text (number, Min: 34, Max: 42) |
| 37 | [pf_fever_method] | Which method of temperature measurement was used by caregiver? *Show the field ONLY if: [pf_fever_pres] = '1'* | checkbox 1 Forehead infrared 2 Axillary 3 Tympanic 4 Other 5 Data not available |
| 38 | [pf_fever_antipy] | Were any antipyretics given at home in the 8 hours before presentation? *This includes any paracetamol or NSAIDS (or brand name equivalents e.g. Calpol, Calprofen)* | radio 1 Yes 2 No 3 Data not available |
| 39 | [pf_contacts] | Were any unwell contacts reported by the caregiver? *This includes any household contacts with symptoms in keeping with infection e.g. fever, respiratory symptoms, gastrointestinal symptoms* | radio 1 Yes 2 No 3 Data not available |
| 40 | [pf_sx] | Which (if any) of the following symptoms were reported by caregivers in the 24 hours preceding presentation? *Select all that apply. This question relates only to symptoms reported by the parent/carer, not findings on examination by a healthcare professional.* | checkbox 1 Cough 2 Coryza 3 Vomiting 4 Diarrhoea 5 Irritability 6 Lethargy 7 Reduced feeding or reluctance to feed 8 Reduced response to social cues 9 Abdominal pain or distension 10 Limb and/or joint swelling 11 Appearing unwell to carer 12 Rash 13 Reduction in urine output |
| 41 | [pf_sx_other] | Please provide details of any further symptoms reported by carer at initial hospital attendance | text |
| 42 | [pf_ex_pres] | Which (if any) of the following findings were reported on examination in the place of initial assessment? | checkbox 1 Pale/mottled/ashen/blue skin, lips or tongue 2 No response to social cues from parents 3 Appearing ill to a healthcare professional 4 Not rousable or if roused does not stay awake 5 Weak, high-pitched or continuous cry 6 Grunting 7 Moderate or severe chest indrawing 8 Reduced skin turgor, skin doughy or tented, sunken eyes 9 Bulging fontanelle 10 Reduced urine output 11 Neck stiffness 12 Seizure/focal neurological signs 13 Non-blanching rash/skin lesions larger than 2mm in diameter 14 Abdominal distension 15 Lethargy 16 Irritability 17 Swelling of a limb/joint 18 No smile or face anxious, dull, expressionless 19 None 20 Other |
| 43 | [pf_ex_presoth] | Please provide details of any further findings on clinical examination | text |
| 44 | [presenting_features_complete] | Complete? | dropdown 0 Incomplete 1 Unverified 2 Complete |

## Vital Signs

| **#** | **Variable Name** | **Field Label / Field Note** | **Field Attributes / Choices** |
| --- | --- | --- | --- |
| 45 | [vs_temp_rec] | Was the infant's temperature measured at initial assessment by a healthcare professional? | radio 1 Yes 2 No 3 Data not available |
| 46 | [vs_temp_initial] | What was the temperature at initial assessment? *Show the field ONLY if: [vs_temp_rec] = '1'* | text (number, Min: 33, Max: 42) |
| 47 | [vs_temp_initialmeth] | How was temperature measured by healthcare professional? *Show the field ONLY if: [vs_temp_rec] = '1'* | radio 1 Forehead infrared 2 Axillary 3 Tympanic 4 Other 5 Data not available |
| 48 | [vs_temp_any] | Was temperature rechecked during the infant's time in place of initial assessment? *Show the field ONLY if: [vs_temp_rec] = '1'. This includes any repeated temperature measurement in the location the infant initially presented (e.g. A&E, assessment unit)* | radio 1 Yes 2 No 3 Data not available |
| 49 | [vs_temp_max] | What was the maximum temperature recorded in place of initial assessment? *Show the field ONLY if: [vs_temp_any] = '1'* | text (number, Min: 33, Max: 42) |
| 50 | [vs_hr_rec] | Was heart rate recorded at initial assessment? | radio 1 Yes 2 No 3 Data not available |
| 51 | [vs_hr_initial] | What was the initial heart rate? *Show the field ONLY if: [vs_hr_rec] = '1'* | text (integer, Min: 60, Max: 320) |
| 52 | [vs_hr_repeat] | Was heart rate rechecked in place of initial assessment? *Show the field ONLY if: [vs_hr_rec] = '1'* | radio 1 Yes 2 No 3 Data not available |
| 53 | [vs_hr_max] | What was the maximum heart rate (in place of initial assessment)? *Show the field ONLY if: [vs_hr_repeat] = '1'* | text (integer, Min: 60, Max: 320) |
| 54 | [vs_bp_rec] | Was blood pressure recorded at initial assessment? | radio 1 Yes 2 No 3 Data not available |
| 55 | [vs_bp_initial] | What was the initial blood pressure? *Show the field ONLY if: [vs_bp_rec] = '1'. Systolic mmHg/diastolic mmHg* | text |
| 56 | [vs_rr_rec] | Was respiratory rate recorded at initial assessment? | radio 1 Yes 2 No 3 Data not available |
| 57 | [vs_rr_initial] | What was the initial respiratory rate? *Show the field ONLY if: [vs_rr_rec] = '1'* | text (integer, Min: 20, Max: 90) |
| 58 | [vs_sat_rec] | Was SpO2 recorded at initial assessment? | radio 1 Yes 2 No 3 Data not available |
| 59 | [vs_sat_initial] | What was the initial SpO2? (%) *Show the field ONLY if: [vs_sat_rec] = '1'* | text (integer, Min: 20, Max: 100) |
| 60 | [vs_sat_ox] | Was the initial SpO2 measured in air or oxygen? *Show the field ONLY if: [vs_sat_rec] = '1'* | radio 1 Air 2 Supplemental oxygen 3 Data not available |
| 61 | [vs_crt_rec] | Was capillary refill time (CRT) recorded at initial assessment? | radio 1 Yes 2 No 3 Data not available |
| 62 | [vs_crt_where] | Where was CRT measured? *Show the field ONLY if: [vs_crt_rec] = '1'* | checkbox 1 Centrally 2 Peripherally 3 Not recorded |
| 63 | [vs_crt_cent] | What was the central CRT? *Show the field ONLY if: [vs_crt_where(1)] = '1'* | radio 1 <= 2 seconds 2 > 2 seconds |
| 64 | [vs_crt_periph] | What was the peripheral CRT? *Show the field ONLY if: [vs_crt_where(2)] = '1'* | radio 1 <= 2 seconds 2 > 2 seconds |
| 65 | [vs_crt_unspec] | What was the CRT (site unspecified)? *Show the field ONLY if: [vs_crt_where(3)] = '1'* | radio 1 <= 2 seconds 2 > 2 seconds |
| 66 | [vital_signs_complete] | Complete? | dropdown 0 Incomplete 1 Unverified 2 Complete |

## Investigations

| **#** | **Variable Name** | **Field Label / Field Note** | **Field Attributes / Choices** |
| --- | --- | --- | --- |
| 67 | [inv_any] | Which of the following investigations were performed during this hospital attendance? | checkbox 1 FBC 2 CRP 3 Other blood tests e.g. ALT, U&Es 4 Blood gas 5 Blood culture 6 Blood serology 7 Blood virology 8 Urine dipstick and/or MC&S 9 CSF MC&S and/or virology 10 Stool culture and/or virology 11 NPA/throat swab 12 SARS-CoV-2 investigations 13 Wound/skin swab 14 CXR 15 Other radiological investigations |
| 68 | [inv_fbc_time] | When was first FBC taken? *Show the field ONLY if: [inv_any(1)] = '1'* | radio 1 Within 1 hour of presentation 2 1-4 hours after presentation 3 4-12 hours after presentation 4 12-24 hours after presentation 5 >24 hours after presentation 6 Data not available |
| 69 | [inv_hb_taken] | Is the haemoglobin result available? *Show the field ONLY if: [inv_any(1)] = '1'* | radio 1 Yes 2 No |
| 70 | [inv_hb_value] | What was the initial haemoglobin level? (Units: g/L) *Show the field ONLY if: [inv_hb_taken] = '1'* | text (number, Min: 20, Max: 250) |
| 71 | [inv_wcc_taken] | Is the WCC result available? *Show the field ONLY if: [inv_any(1)] = '1'* | radio 1 Yes 2 No 3 Data not available |
| 72 | [inv_wcc_first] | First WCC value (Units: 10^9/L) *Show the field ONLY if: [inv_wcc_taken] = '1'* | text (number, Min: 0, Max: 50) |
| 73 | [inv_wcc_repeat] | Was the WCC repeated? *Show the field ONLY if: [inv_wcc_taken] = '1'* | radio 1 Yes 2 No 3 Data not available |
| 74 | [inv_wcc_peak] | Peak WCC value (Units: 10^9/L) *Show the field ONLY if: [inv_wcc_repeat] = '1'* | text (number, Min: 0, Max: 50) |
| 75 | [inv_neut_taken] | Is the neutrophil count available? *Show the field ONLY if: [inv_any(1)] = '1'* | radio 1 Yes 2 No 3 Data not available |
| 76 | [inv_neut_first] | First neutrophil value (Units: 10^9/L) *Show the field ONLY if: [inv_neut_taken] = '1'* | text (number, Min: 0, Max: 30) |
| 77 | [inv_neut_repeat] | Was the neutrophil count repeated? *Show the field ONLY if: [inv_neut_taken] = '1'* | radio 1 Yes 2 No 3 Data not available |
| 78 | [inv_neut_peak] | Peak neutrophil value (Units: 10^9/L) *Show the field ONLY if: [inv_neut_repeat] = '1'* | text (number, Min: 0, Max: 30) |
| 79 | [inv_lymp_taken] | Is the lymphocyte count available? *Show the field ONLY if: [inv_any(1)] = '1'* | radio 1 Yes 2 No 3 Data not available |
| 80 | [inv_lymp_first] | First lymphocyte value (Units: 10^9/L) *Show the field ONLY if: [inv_lymp_taken] = '1'* | text (number, Min: 0, Max: 30) |
| 81 | [inv_lymp_repeat] | Was the lymphocyte count repeated? *Show the field ONLY if: [inv_lymp_taken] = '1'* | radio 1 Yes 2 No 3 Data not available |
| 82 | [inv_lymp_peak] | Peak lymphocyte value (Units: 10^9/L) *Show the field ONLY if: [inv_lymp_repeat] = '1'* | text (number, Min: 0, Max: 30) |
| 83 | [inv_crp_taken] | Is the CRP result available? *Show the field ONLY if: [inv_any(2)] = '1'* | radio 1 Yes 2 No |
| 84 | [inv_crp_time] | When was the first CRP taken? *Show the field ONLY if: [inv_crp_taken] = '1'* | radio 1 Within 1 hour of presentation 2 1-4 hours after presentation 3 4-12 hours after presentation 4 12-24 hours after presentation 5 >24 hours after presentation 6 Data not available |
| 85 | [inv_crp_first] | What was the initial CRP value? (Units: mg/L) *Show the field ONLY if: [inv_crp_taken] = '1'. If CRP value is recorded as <1, enter 0* | text (number, Min: 0, Max: 400) |
| 86 | [inv_crp_serial] | Were serial CRPs taken? *Show the field ONLY if: [inv_crp_taken] = '1'* | radio 1 Yes 2 No 3 Data not available |
| 87 | [inv_crp_peak] | What was the peak CRP value? (Units: mg/L) *Show the field ONLY if: [inv_crp_serial] = '1'* | text (number, Min: 1, Max: 300) |
| 88 | [inv_alt_taken] | Was an ALT taken? *Show the field ONLY if: [inv_any(3)] = '1'* | radio 1 Yes 2 No |
| 89 | [inv_alt_first] | What was the first ALT value? (Units: iU/L) *Show the field ONLY if: [inv_alt_taken] = '1'* | text (number, Min: 7, Max: 10000) |
| 90 | [inv_creat_taken] | Was a creatinine taken? *Show the field ONLY if: [inv_any(3)] = '1'* | radio 1 Yes 2 No |
| 91 | [inv_creat_first] | What was the initial creatinine value? *Show the field ONLY if: [inv_creat_taken] = '1'* | text (number, Min: 5, Max: 300) |
| 92 | [inv_lac_taken] | Is the lactate value available? *Show the field ONLY if: [inv_any(4)] = '1'* | radio 1 Yes 2 No |
| 93 | [inv_lac_time] | When was the lactate taken? *Show the field ONLY if: [inv_lac_taken] = '1'* | radio 1 Within 1 hour of presentation 2 1-4 hours after presentation 3 4-12 hours after presentation 4 12-24 hours after presentation 5 >24 hours after presentation 6 Data not available |
| 94 | [inv_lac_method] | How was the lactate taken? *Show the field ONLY if: [inv_lac_taken] = '1'* | radio 1 Capillary 2 Venous 3 Other 4 Data not available |
| 95 | [inv_lac_first] | What was the initial lactate? (Units: mmol/L) *Show the field ONLY if: [inv_lac_taken] = '1'* | text (number, Min: 0, Max: 30) |
| 96 | [inv_bc_taken] | Are blood culture results available? *Show the field ONLY if: [inv_any(5)] = '1'* | radio 1 Yes 2 No |
| 97 | [inv_bc_date] | When was the first blood culture taken? *Show the field ONLY if: [inv_bc_taken] = '1'* | text (datetime_ymd) |
| 98 | [inv_bc_abx] | When was the blood culture taken in relation to antibiotic administration? *Show the field ONLY if: [inv_bc_taken] = '1'* | radio 1 Before antibiotics were given 2 After antibiotics were given 3 Data not available |
| 99 | [inv_bc_result] | What was the result of the blood culture? *Show the field ONLY if: [inv_bc_taken] = '1'* | radio 1 Positive 2 Negative 3 Data not available |
| 100 | [inv_bc_org] | Which organisms were present in the blood culture? *Show the field ONLY if: [inv_bc_taken] = '1' and [inv_bc_result] = '1'* | checkbox 1 Coagulase-negative streptococcus 2 Streptococcus pneumoniae 3 Haemophilus influenzae 4 Escherichia coli 5 Enterobacter spp 6 Enterococcus spp 7 Staphylococcus aureus 8 Group B streptococcus 9 Klebsiella 10 Other 11 Data not available |
| 101 | [inv_bc_oth] | Please provide further details of the blood culture result *Show the field ONLY if: [inv_bc_taken] = '1' and [inv_bc_result] = '1'* | text |
| 102 | [inv_bc_contam] | Was the blood culture result treated as contaminant? *Show the field ONLY if: [inv_bc_taken] = '1'* | radio 1 Yes 2 No 3 Data not available |
| 103 | [inv_sero_taken] | Are serological test results available? *Show the field ONLY if: [inv_any(6)] = '1'. Serological tests include but are not limited to specific immunoglobulins e.g. EBV or CMV IgM/IgG* | radio 1 Yes 2 No |
| 104 | [inv_sero_tests] | Which serological tests were performed? *Show the field ONLY if: [inv_sero_taken] = '1'* | text |
| 105 | [inv_sero_results] | What were the results of this test? *Show the field ONLY if: [inv_sero_taken] = '1'* | text |
| 106 | [inv_viro_taken] | Are virological blood test results available? *Show the field ONLY if: [inv_any(7)] = '1'. Virological tests include but are not limited to viral PCR performed on blood e.g. HSV PCR. This does not include virological tests performed on other samples e.g. NPA or CSF.* | radio 1 Yes 2 No 3 Data not available |
| 107 | [inv_viro_tests] | Which virological tests were performed? *Show the field ONLY if: [inv_viro_taken] = '1'* | text |
| 108 | [inv_viro_results] | What were the results of this test? *Show the field ONLY if: [inv_viro_taken] = '1'* | text |
| 109 | [inv_ur_taken] | Is the urine sample result available? *Show the field ONLY if: [inv_any(8)] = '1'* | radio 1 Yes 2 No |
| 110 | [inv_ur_meth] | How was the urine sample collected? *Show the field ONLY if: [inv_ur_taken] = '1'* | radio 1 Clean catch 2 Bag 3 Catheter 4 Suprapubic aspirate 5 Other 6 Data not available |
| 111 | [inv_ur_abx] | When was the first urine sample collected in relation to antibiotic administration? *Show the field ONLY if: [inv_ur_taken] = '1'* | radio 1 Before antibiotics were given 2 After antibiotics were given 3 Data not available |
| 112 | [inv_ur_dip] | Was a urine dip performed? *Show the field ONLY if: [inv_ur_taken] = '1'* | radio 1 Yes 2 No 3 Data not available |
| 113 | [inv_ur_leu] | Leucocyte result *Show the field ONLY if: [inv_ur_taken] = '1' and [inv_ur_dip] = '1'* | radio 1 Negative 2 Trace 3 + 4 ++ 5 +++ 6 Data not available |
| 114 | [inv_ur_nit] | Nitrite result *Show the field ONLY if: [inv_ur_taken] = '1' and [inv_ur_dip] = '1'* | radio 1 Negative 2 Trace 3 Positive 4 Data not available |
| 115 | [inv_ur_mcs] | Was the urine sample sent for MC&S? *Show the field ONLY if: [inv_ur_taken] = '1'* | radio 1 Yes 2 No 3 Data not available |
| 116 | [inv_ur_wcc] | Urine microscopy WCC (Units: WBC/uL) *Show the field ONLY if: [inv_ur_taken] = '1' and [inv_ur_mcs] = '1'* | radio 1 <10 2 10-100 3 =>100 4 Data not available |
| 117 | [inv_ur_rbc] | Urine microscopy RBC (Units: RBC/uL) *Show the field ONLY if: [inv_ur_taken] = '1' and [inv_ur_mcs] = '1'* | radio 1 0-5 2 5-25 3 =>25 4 Data not available |
| 118 | [inv_ur_bac] | Urine microscopy bacteria count *Show the field ONLY if: [inv_ur_taken] = '1' and [inv_ur_mcs] = '1'* | radio 1 <=9,999 2 10,000-49,999 3 50,000-99,999 4 =>100,000 5 Data not available |
| 119 | [inv_ur_org] | Which organisms were identified on MC&S? *Show the field ONLY if: [inv_ur_taken] = '1' and [inv_ur_mcs] = '1'* | checkbox 1 Escherichia coli 2 Proteus spp 3 Enterococcus 4 Klebsiella 5 Moraxella catarrhalis 6 Pseudomonas aeruginosa 7 Enterobacter spp 8 Enterococcus spp 9 Other 10 Mixed growth 11 None/negative 12 Data not available |
| 120 | [inv_ur_orgadd] | Please provide further details of urine MC&S *Show the field ONLY if: [inv_ur_taken] = '1' and [inv_ur_mcs] = '1'* | text |
| 121 | [inv_ur_contam] | Was the urine MC&S result treated as a contaminant? *Show the field ONLY if: [inv_ur_taken] = '1' and [inv_ur_mcs] = '1'* | radio 1 Yes 2 No 3 Data not available |
| 122 | [inv_ur_multi] | Were multiple urine samples taken? *Show the field ONLY if: [inv_ur_taken] = '1'* | radio 1 Yes 2 No 3 Data not available |
| 123 | [inv_csf_taken] | Is the result of the lumbar puncture available? *Show the field ONLY if: [inv_any(9)] = '1'* | radio 1 Yes 2 No |
| 124 | [inv_csf_abx] | When were CSF samples obtained in relation to antibiotic administration? *Show the field ONLY if: [inv_csf_taken] = '1'* | radio 1 Before antibiotics were given 2 After antibiotics were given 3 Data not available |
| 125 | [inv_csf_wcc] | What was the CSF WCC? (Units: cells/uL) *Show the field ONLY if: [inv_csf_taken] = '1'* | text (number) |
| 126 | [inv_csf_rbc] | What was the CSF RBC? (Units: cells/mm3) *Show the field ONLY if: [inv_csf_taken] = '1'* | text (number) |
| 127 | [inv_csf_gluc] | What was the CSF glucose? (Units: mmol/L) *Show the field ONLY if: [inv_csf_taken] = '1'* | text (number, Min: 0, Max: 5) |
| 128 | [inv_csf_pairedgluc] | What was the paired blood glucose? (Units: mmol/L) *Show the field ONLY if: [inv_csf_taken] = '1'* | text (number, Min: 0, Max: 10) |
| 129 | [inv_csf_prot] | What was the CSF protein? (Units: g/L) *Show the field ONLY if: [inv_csf_taken] = '1'* | text (number) |
| 130 | [inv_csf_gram] | What was the CSF Gram stain result? *Show the field ONLY if: [inv_csf_taken] = '1'* | radio 1 Positive 2 Negative 3 Data not available |
| 131 | [inv_csf_mcs] | What was the CSF MC&S result? *Show the field ONLY if: [inv_csf_taken] = '1'* | radio 1 Positive 2 Negative 3 Data not available |
| 132 | [inv_csf_bacorg] | Which bacterial organisms were identified on CSF MC&S? *Show the field ONLY if: [inv_csf_mcs] = '1'* | checkbox 1 Streptococcus pneumoniae 2 Haemophilus influenzae 3 Escherichia coli 4 Neisseria meningitidis 5 Listeria meningitidis 6 Group B streptococcus 7 Other 8 Data not available |
| 133 | [inv_csf_bacoth] | Please provide further details of bacterial organisms identified in CSF samples *Show the field ONLY if: [inv_csf_mcs] = '1'* | text |
| 134 | [inv_csf_contam] | Was the CSF sample treated as a contaminant? *Show the field ONLY if: [inv_csf_mcs] = '1'* | radio 1 Yes 2 No 3 Data not available |
| 135 | [inv_csf_virosent] | Was CSF sent for virology PCR? *Show the field ONLY if: [inv_csf_taken] = '1'* | radio 1 Yes 2 No 3 Data not available |
| 136 | [inv_csf_virores] | What was the CSF virology PCR result? *Show the field ONLY if: [inv_csf_virosent] = '1'* | radio 1 Positive 2 Negative 3 Data not available |
| 137 | [inv_csf_viroorg] | Which viral organisms were identified in the CSF sample? *Show the field ONLY if: [inv_csf_virores] = '1'* | checkbox 1 Enterovirus 2 Rhinovirus 3 HSV 4 VZV 5 Parechovirus 6 Other 7 Data not available |
| 138 | [inv_csf_virooth] | Please provide further details of viral organisms identified in CSF samples *Show the field ONLY if: [inv_csf_virores] = '1'* | text |
| 139 | [inv_st_taken] | Is the result of the stool sample available? *Show the field ONLY if: [inv_any(10)] = '1'* | radio 1 Yes 2 No |
| 140 | [inv_st_res] | What was the stool culture result? *Show the field ONLY if: [inv_st_taken] = '1'* | radio 1 Positive 2 Negative 3 Data not available |
| 141 | [inv_st_bac] | Which (if any) bacterial organisms were found on stool culture? *Show the field ONLY if: [inv_st_taken] = '1' and [inv_st_res] = '1'* | checkbox 1 Ova present 2 Cysts present 3 Parasites present 4 Campylobacter jejuni 5 Clostridium difficile 6 Other 7 None 8 Data not available |
| 142 | [inv_st_vir] | What (if any) viral organisms were found on stool testing? *Show the field ONLY if: [inv_st_taken] = '1' and [inv_st_res] = '1'* | checkbox 1 Enterovirus 2 Adenovirus 3 Rotavirus 4 Norovirus 5 Other 6 None 7 Data not available |
| 143 | [inv_st_oth] | Please provide further details of the stool culture and/or virology result *Show the field ONLY if: [inv_st_taken] = '1' and [inv_st_res] = '1'* | text |
| 144 | [inv_npa_taken] | Is the NPA result available? *Show the field ONLY if: [inv_any(11)] = '1'* | radio 1 Yes 2 No 3 Data not available |
| 145 | [inv_npa_res] | What was the result of the NPA? *Show the field ONLY if: [inv_npa_taken] = '1'* | radio 1 Positive 2 Negative 3 Data not available |
| 146 | [inv_npa_vir] | Which viral organisms were identified on NPA? *Show the field ONLY if: [inv_npa_res] = '1'* | checkbox 1 Rhino/enterovirus 2 Parainfluenza 1 3 Parainfluenza 2 4 Parainfluenza 3 5 Parainfluenza 4 6 Adenovirus 7 Bocavirus 8 Coronaviruses (not Covid-19) 9 Metapneumovirus 10 Parechovirus 11 RSV A 12 RSV B 13 Influenza A 14 Influenza B 15 Other 16 None 17 Data not available |
| 147 | [inv_npa_viroth] | Please provide further details of viral organisms identified in NPA samples *Show the field ONLY if: [inv_npa_res] = '1'* | text |
| 148 | [inv_npa_bac] | Which bacterial organisms were identified on NPA? *Show the field ONLY if: [inv_npa_res] = '1'* | checkbox 1 Bordatella pertussis 2 Mycoplasma pneumoniae 3 Streptococcal pneumoniae 4 Haemophilus influenzae 5 Other 6 None 7 Data not available |
| 149 | [inv_npa_bacoth] | Please provide further details of bacterial organisms identified in NPA samples *Show the field ONLY if: [inv_npa_res] = '1'* | text |
| 150 | [inv_cov_taken] | Is the result for SARS-CoV-2 testing available? *Show the field ONLY if: [inv_any(12)] = '1'. This includes any type of SARS-CoV-2 test e.g. lateral flow, PCR and serology* | radio 1 Yes 2 No |
| 151 | [inv_cov_lftaken] | Was a SARS-CoV-2 lateral flow taken? *Show the field ONLY if: [inv_cov_taken] = '1'* | radio 1 Yes 2 No 3 Data not available |
| 152 | [inv_cov_lfres] | What was the SARS-CoV-2 lateral flow result? *Show the field ONLY if: [inv_cov_lftaken] = '1'* | radio 1 Positive 2 Negative 3 Data not available |
| 153 | [inv_cov_pcrtaken] | Was a SARS-CoV-2 PCR taken? *Show the field ONLY if: [inv_cov_taken] = '1'* | radio 1 Yes 2 No 3 Data not available |
| 154 | [inv_cov_pcrres] | What was the SARS-CoV-2 PCR result? *Show the field ONLY if: [inv_cov_pcrtaken] = '1'* | radio 1 Positive 2 Negative 3 Data not available |
| 155 | [inv_cov_sertaken] | Was SARS-CoV-2 serology taken? *Show the field ONLY if: [inv_cov_taken] = '1'* | radio 1 Yes 2 No 3 Data not available |
| 156 | [inv_cov_serres] | What was the SARS-CoV-2 serology result? *Show the field ONLY if: [inv_cov_sertaken] = '1'* | radio 1 Positive 2 Negative 3 Data not available |
| 157 | [inv_sk_taken] | Is the result of the wound and/or skin swab available? *Show the field ONLY if: [inv_any(13)] = '1'* | radio 1 Yes 2 No 3 Data not available |
| 158 | [inv_sk_res] | What was the wound/skin swab result? *Show the field ONLY if: [inv_sk_taken] = '1'* | radio 1 Positive 2 Negative 3 Data not available |
| 159 | [inv_sk_org] | Which organism was identified on wound/skin swab? *Show the field ONLY if: [inv_sk_res] = '1'* | checkbox 1 Skin flora 2 Staphylococcus aureus 3 Methicillin resistant staphylococcus aureus 4 Herpes simplex 5 Other |
| 160 | [inv_sk_orgoth] | Please provide further details of the wound/skin swab result *Show the field ONLY if: [inv_sk_res] = '1'* | text |
| 161 | [inv_cxr_taken] | Is the result of the CXR available? *Show the field ONLY if: [inv_any(14)] = '1'. Imaging should relate only to radiological investigations performed in relation to the infant's initial presentation with fever and not to any secondary complications or unrelated issues.* | radio 1 Yes 2 No |
| 162 | [inv_cxr_norm] | Was the CXR normal or abnormal? *Show the field ONLY if: [inv_cxr_taken] = '1'* | radio 1 Normal 2 Abnormal 3 Data not available |
| 163 | [inv_cxr_res] | What was the CXR result? *Show the field ONLY if: [inv_cxr_norm] = '2'* | checkbox 1 Consolidation 2 Bronchial hyperreactivity or perihilar changes 3 Empyema 4 Effusion 5 Pneumothorax 6 Other 7 Data not available |
| 164 | [inv_cxr_oth] | Please provide further details of the CXR result *Show the field ONLY if: [inv_cxr_norm] = '2'* | text |
| 165 | [inv_us_taken] | Was an ultrasound performed? *Show the field ONLY if: [inv_any(15)] = '1'. Imaging should relate only to radiological investigations performed in relation to the infant's initial presentation with fever and not to any secondary complications or unrelated issues.* | radio 1 Yes 2 No 3 Data not available |
| 166 | [inv_us_type] | What type of ultrasound was performed? *Show the field ONLY if: [inv_us_taken] = '1'. Select all that apply* | checkbox 1 Chest 2 Abdomen 3 Kidneys 4 Head 5 Other 6 Data not available |
| 167 | [inv_us_res] | What was the result of the ultrasound? *Show the field ONLY if: [inv_us_taken] = '1'* | radio 1 Normal 2 Abnormal 3 Data not available |
| 168 | [inv_us_det] | Please provide further details of ultrasound report(s) *Show the field ONLY if: [inv_us_taken] = '1'* | text |
| 169 | [inv_ct_taken] | Was a CT scan performed? *Show the field ONLY if: [inv_any(15)] = '1'. Imaging should relate only to radiological investigations performed in relation to the infant's initial presentation with fever and not to any secondary complications or unrelated issues.* | radio 1 Yes 2 No 3 Data not available |
| 170 | [inv_ct_type] | What type of CT scan was performed? *Show the field ONLY if: [inv_ct_taken] = '1'. Please select all that apply* | checkbox 1 Chest 2 Abdomen 3 Kidneys 4 Head 5 Other 6 Data not available |
| 171 | [inv_ct_res] | What was the result of the CT scan? *Show the field ONLY if: [inv_ct_taken] = '1'* | radio 1 Normal 2 Abnormal 3 Data not available |
| 172 | [inv_ct_det] | Please provide further details of the CT scan report(s) *Show the field ONLY if: [inv_ct_taken] = '1'* | text |
| 173 | [inv_mri_taken] | Was an MRI scan performed? *Show the field ONLY if: [inv_any(15)] = '1'. Imaging should relate only to radiological investigations performed in relation to the infant's initial presentation with fever and not to any secondary complications or unrelated issues.* | radio 1 Yes 2 No 3 Data not available |
| 174 | [inv_mri_type] | What type of MRI scan was performed? *Show the field ONLY if: [inv_mri_taken] = '1'. Please select all that apply* | checkbox 1 Chest 2 Abdomen 3 Kidneys 4 Head 5 Other 6 Data not available |
| 175 | [inv_mri_res] | What was the result of the MRI scan? *Show the field ONLY if: [inv_mri_taken] = '1'* | radio 1 Normal 2 Abnormal 3 Data not available |
| 176 | [inv_mri_det] | Please provide further details of the MRI scan report(s) *Show the field ONLY if: [inv_mri_taken] = '1'* | text |
| 177 | [investigations_complete] | Complete? | dropdown 0 Incomplete 1 Unverified 2 Complete |

## Management

| **#** | **Variable Name** | **Field Label / Field Note** | **Field Attributes / Choices** |
| --- | --- | --- | --- |
| 178 | [man_regabx_any] | Was the infant receiving regular antimicrobials prior to hospital attendance? | radio 1 Yes 2 No 3 Data not available |
| 179 | [man_acuteabx_any] | Were any antibiotics given acutely in the 48 hours prior to hospital stay? | radio 1 Yes 2 No 3 Data not available |
| 180 | [man_acuteabx_where] | Where was the decision made to start antibiotics in the 48 hours prior to hospital attendance? *Show the field ONLY if: [man_acuteabx_any] = '1'* | radio 1 Community 2 Hospital 3 Other 4 Data not available |
| 181 | [man_acuteabx_type] | Which antibiotics had been prescribed? *Show the field ONLY if: [man_acuteabx_any] = '1'* | checkbox 1 Amoxicillin 2 Co-amoxiclav 3 Sulfamethoxazole/trimethoprim 4 Macrolide (please give further details) 5 Cephalosporin (please give further details) 6 Flucloxacillin 7 Penicillin or benzylpenicillin V 8 Gentamicin 9 Clindamycin 10 Nitrofurantoin 11 Vancomycin 12 Other (please give further details) 13 Data not available |
| 182 | [man_acuteabx_further] | Please provide further details of antibiotic prescribed prior to hospital attendance *Show the field ONLY if: [man_acuteabx_any] = '1'* | text |
| 183 | [man_acuteabx_route] | Route of administration of antibiotic prescribed in the 48 hours prior to hospital attendance *Show the field ONLY if: [man_acuteabx_any] = '1'* | radio 1 IV 2 PO 3 IM 4 Other 5 Data not available |
| 184 | [man_ipabx_any] | Were any antibiotics prescribed during hospital attendance? | radio 1 Yes 2 No 3 Data not available |
| 185 | [man_ipabx_where] | Where was the decision made to start antibiotics? *Show the field ONLY if: [man_ipabx_any] = '1'* | radio 1 Emergency department 2 Inpatient setting 3 Other 4 Data not available |
| 186 | [man_ipabx_when] | What was the date and time of first antibiotic administration? *Show the field ONLY if: [man_ipabx_any] = '1'* | text (datetime_ymd) |
| 187 | [man_ipabx_which] | Which of the following antibiotics were given during hospital attendance? *Show the field ONLY if: [man_ipabx_any] = '1'* | checkbox 1 Amoxicillin 2 Co-amoxiclav 3 Sulfamethoxazole/trimethoprim 4 Macrolide (any) 5 Cephalosporin (any) 6 Flucloxacillin 7 Penicillin or benzylpenicillin V 8 Gentamicin 9 Clindamycin 10 Nitrofurantoin 11 Trimethoprim 12 Vancomycin 13 Other |
| 188 | [man_amox_totdur] | Duration of course of amoxicillin (Days) *Show the field ONLY if: [man_ipabx_which(1)] = '1'* | text |
| 189 | [man_amox_route] | Route of administration of amoxicillin *Show the field ONLY if: [man_ipabx_which(1)] = '1'* | checkbox 1 IV 2 PO 3 IM 4 Other 5 Data not available |
| 190 | [man_amox_add] | Further details of amoxicillin *Show the field ONLY if: [man_ipabx_which(1)] = '1'* | text |
| 191 | [man_coam_totdur] | Duration of course of co-amoxiclav (Days) *Show the field ONLY if: [man_ipabx_which(2)] = '1'* | text |
| 192 | [man_coam_route] | Route of administration of co-amoxiclav *Show the field ONLY if: [man_ipabx_which(2)] = '1'* | checkbox 1 IV 2 PO 3 IM 4 Other 5 Data not available |
| 193 | [man_coam_add] | Further details of co-amoxiclav *Show the field ONLY if: [man_ipabx_which(2)] = '1'* | text |
| 194 | [man_sept_totdur] | Duration of course of Septrin (Days) *Show the field ONLY if: [man_ipabx_which(3)] = '1'* | text |
| 195 | [man_sept_route] | Route of administration of Septrin *Show the field ONLY if: [man_ipabx_which(3)] = '1'* | checkbox 1 IV 2 PO 3 IM 4 Other 5 Data not available |
| 196 | [man_sept_add] | Further details of antibiotic Septrin *Show the field ONLY if: [man_ipabx_which(3)] = '1'* | text |
| 197 | [man_macr_totdur] | Duration of course of macrolide (Days) *Show the field ONLY if: [man_ipabx_which(4)] = '1'* | text |
| 198 | [man_macr_route] | Route of administration of macrolide *Show the field ONLY if: [man_ipabx_which(4)] = '1'* | checkbox 1 IV 2 PO 3 IM 4 Other 5 Data not available |
| 199 | [man_macr_add] | Further details of macrolide *Show the field ONLY if: [man_ipabx_which(4)] = '1'* | text |
| 200 | [man_ceph_totdur] | Duration of course of cephalosporin (Days) *Show the field ONLY if: [man_ipabx_which(5)] = '1'* | text |
| 201 | [man_ceph_route] | Route of administration of cephalosporin *Show the field ONLY if: [man_ipabx_which(5)] = '1'* | checkbox 1 IV 2 PO 3 IM 4 Other 5 Data not available |
| 202 | [man_ceph_add] | Further details of cephalosporin *Show the field ONLY if: [man_ipabx_which(5)] = '1'* | text |
| 203 | [man_fluc_totdur] | Duration of course of flucloxacillin (Days) *Show the field ONLY if: [man_ipabx_which(6)] = '1'* | text |
| 204 | [man_fluc_route] | Route of administration of flucloxacillin *Show the field ONLY if: [man_ipabx_which(6)] = '1'* | checkbox 1 IV 2 PO 3 IM 4 Other 5 Data not available |
| 205 | [man_fluc_add] | Further details of flucloxacillin *Show the field ONLY if: [man_ipabx_which(6)] = '1'* | text |
| 206 | [man_penc_totdur] | Duration of course of benzylpenicillin/penicillin V (Days) *Show the field ONLY if: [man_ipabx_which(7)] = '1'* | text |
| 207 | [man_penc_route] | Route of administration of benzylpenicillin/penicillin V *Show the field ONLY if: [man_ipabx_which(7)] = '1'* | checkbox 1 IV 2 PO 3 IM 4 Other 5 Data not available |
| 208 | [man_penc_add] | Further details of benzylpenicillin/penicillin V *Show the field ONLY if: [man_ipabx_which(7)] = '1'* | text |
| 209 | [man_gent_totdur] | Duration of course of gentamicin (Days) *Show the field ONLY if: [man_ipabx_which(8)] = '1'* | text |
| 210 | [man_gent_route] | Route of administration of gentamicin *Show the field ONLY if: [man_ipabx_which(8)] = '1'* | checkbox 1 IV 2 PO 3 IM 4 Other 5 Data not available |
| 211 | [man_gent_add] | Further details of gentamicin *Show the field ONLY if: [man_ipabx_which(8)] = '1'* | text |
| 212 | [man_clin_totdur] | Duration of course of clindamycin (Days) *Show the field ONLY if: [man_ipabx_which(9)] = '1'* | text |
| 213 | [man_clin_route] | Route of administration of clindamycin *Show the field ONLY if: [man_ipabx_which(9)] = '1'* | checkbox 1 IV 2 PO 3 IM 4 Other 5 Data not available |
| 214 | [man_clin_add] | Further details of clindamycin *Show the field ONLY if: [man_ipabx_which(9)] = '1'* | text |
| 215 | [man_nitr_totdur] | Duration of course of nitrofurantoin (Days) *Show the field ONLY if: [man_ipabx_which(10)] = '1'* | text |
| 216 | [man_nitr_add] | Further details of nitrofurantoin *Show the field ONLY if: [man_ipabx_which(10)] = '1'* | text |
| 217 | [man_trim_totdur] | Duration of course of trimethoprim (Days) *Show the field ONLY if: [man_ipabx_which(11)] = '1'* | text |
| 218 | [man_trim_add] | Further details of trimethoprim *Show the field ONLY if: [man_ipabx_which(11)] = '1'* | text |
| 219 | [man_vanc_totdur] | Duration of course of vancomycin (Days) *Show the field ONLY if: [man_ipabx_which(12)] = '1'* | text |
| 220 | [man_vanc_route] | Route of administration of vancomycin *Show the field ONLY if: [man_ipabx_which(12)] = '1'* | checkbox 1 IV 2 PO 3 IM 4 Other 5 Data not available |
| 221 | [man_vanc_add] | Further details of vancomycin *Show the field ONLY if: [man_ipabx_which(12)] = '1'* | text |
| 222 | [man_ipabx_adddet] | Further details of additional antibiotics *Show the field ONLY if: [man_ipabx_which(13)] = '1'* | text |
| 223 | [man_ipabx_totalcourse] | Total duration of inpatient antibiotic course (Days) *Show the field ONLY if: [man_ipabx_any] = '1'. Combined course of all inpatient antibiotics related to the initial presentation in days* | text |
| 224 | [man_ipav_any] | Were any antivirals given during the hospital attendance? | radio 1 Yes 2 No 3 Data not available |
| 225 | [man_ipav_where] | Where was the decision made to start antivirals? *Show the field ONLY if: [man_ipav_any] = '1'* | radio 1 Emergency department 2 Inpatient setting 3 Other 4 Data not available |
| 226 | [man_ipav_which] | Which of the following antivirals were given during hospital attendance? *Show the field ONLY if: [man_ipav_any] = '1'* | checkbox 1 Aciclovir 2 Valganciclovir 3 Tamiflu/oseltamivir 4 Remdesevir 5 Other |
| 227 | [man_acic_route] | Route of aciclovir course *Show the field ONLY if: [man_ipav_which(1)] = '1'* | radio 1 IV 2 PO 3 IM 4 Other 5 Data not available |
| 228 | [man_acic_totdur] | Total duration of aciclovir course *Show the field ONLY if: [man_ipav_which(1)] = '1'* | text |
| 229 | [man_valg_route] | Route of valganciclovir course *Show the field ONLY if: [man_ipav_which(2)] = '1'* | radio 1 IV 2 PO 3 IM 4 Other 5 Data not available |
| 230 | [man_valg_totdur] | Total duration of valganciclovir course *Show the field ONLY if: [man_ipav_which(2)] = '1'* | text |
| 231 | [man_tami_totdur] | Total duration of Tamiflu course *Show the field ONLY if: [man_ipav_which(3)] = '1'* | text |
| 232 | [man_remdes_totdur] | Total duration of Remdesevir course *Show the field ONLY if: [man_ipav_which(4)] = '1'* | text |
| 233 | [man_avoth_det] | Further details of other antivirals *Show the field ONLY if: [man_ipav_which(5)] = '1'* | text |
| 234 | [man_avoth_route] | Route of other antiviral course *Show the field ONLY if: [man_ipav_which(5)] = '1'* | radio 1 IV 2 PO 3 IM 4 Other 5 Data not available |
| 235 | [man_avoth_totdur] | Total duration of other antiviral course *Show the field ONLY if: [man_ipav_which(5)] = '1'* | text |
| 236 | [man_av_totalcourse] | Total course of inpatient antiviral course (Days) *Show the field ONLY if: [man_ipav_any] = '1'. Combined course of all inpatient antivirals related to the initial presentation in days* | text |
| 237 | [man_flu_rec] | Did the infant receive a fluid bolus during the first 6 hours of presentation? | radio 1 Yes 2 No 3 Data not available |
| 238 | [man_flu_vol] | What was the cumulative volume of fluid boluses given in the first 6 hours? *Show the field ONLY if: [man_flu_rec] = '1'* | radio 1 10ml/kg 2 20ml/kg 3 30ml/kg 4 40ml/kg 5 Other 6 Data not available |
| 239 | [man_add_ox] | Did the infant receive any supplemental oxygen at any point during this hospital attendance? | radio 1 Yes 2 No 3 Data not available |
| 240 | [man_add_resp] | Did the infant receive any respiratory support during this hospital attendance? | radio 1 Yes 2 No 3 Data not available |
| 241 | [man_add_resptype] | What type(s) of respiratory support was given? *Show the field ONLY if: [man_add_resp] = '1'. Select all that apply* | checkbox 1 High-flow oxygen 2 CPAP 3 Intubation and invasive ventilation 4 Other 5 Data not available |
| 242 | [man_add_ino] | Did the infant receive any inotropes during this hospital admission? | radio 1 Yes 2 No 3 Data not available |
| 243 | [management_complete] | Complete? | dropdown 0 Incomplete 1 Unverified 2 Complete |

## Outcomes

| **#** | **Variable Name** | **Field Label / Field Note** | **Field Attributes / Choices** |
| --- | --- | --- | --- |
| 244 | [out_work_diag] | What was the working diagnosis after initial assessment during this hospital attendance? | checkbox 1 Upper respiratory tract infection 2 Lower respiratory tract infection 3 Gastro-intestinal infection 4 Urinary tract infection 5 Childhood exanthem 6 Soft tissue or skin infection 7 Musculoskeletal infection 8 Sepsis 9 Meningitis or other CNS infection 10 Inflammatory illness 11 Undifferentiated fever 12 Surgical abdomen 13 Other 14 Data not available |
| 245 | [out_work_add] | Working diagnosis - additional information | text |
| 246 | [out_trans_where] | What was the outcome after initial assessment? | radio 1 Discharged home from place of initial assessment 2 Admission to an inpatient ward 3 Admission to a clinical decision unit or equivalent 4 Transferred to a paediatric assessment unit or equivalent 5 Ambulated from an inpatient setting 6 Discharged home with hospital at home service 7 Admitted to PICU 8 Transferred via critical care retrieval service 9 Transferred to another hospital (non-critical care transfer) 10 Transferred to theatre 11 Other 12 Data not available |
| 247 | [out_trans_rep] | If discharged home, did the infant represent to an emergency setting in the following 5 days? *Show the field ONLY if: [out_trans_where] = '1'* | radio 1 Yes 2 No 3 Data not available |
| 248 | [out_trans_date] | Date and time of transfer/admission/discharge from place of initial assessment | text (datetime_ymd) |
| 249 | [out_trans_subs] | What was the highest level of care required during this hospital attendance? | radio 1 Inpatient ward 2 HDU 3 ICU 4 Anaesthetics/critical care retrieval team support prior to transfer 5 None - discharged from place of initial assessment 6 Data not available |
| 250 | [out_disc_date] | Date and time of discharge from hospital | text (datetime_ymd) |
| 251 | [out_disc_fup] | Were there any hospital follow-up plans made at discharge? *This includes any hospital-based follow-up including at other hospitals to the location the infant initially presented at (e.g. if the infant initially presented to a hospital other than their local). This does not include follow-up in primary care.* | radio 1 Yes 2 No 3 Data not available |
| 252 | [out_disc_fupdet] | Details of hospital follow-up plans *Show the field ONLY if: [out_disc_fup] = '1'* | text |
| 253 | [out_disc_dur] | What was the duration of hospital stay? | radio 1 <24 hours 2 24-48 hours 3 48-72 hours 4 >72 hours 5 Data not available |
| 254 | [out_disc_diag] | What was the diagnosis at discharge? | checkbox 1 Upper respiratory tract infection 2 Lower respiratory tract infection 3 Gastro-intestinal infection 4 Urinary tract infection 5 Childhood exanthem 6 Soft tissue or skin infection 7 Musculoskeletal infection 8 Sepsis 9 Meningitis or other CNS infection 10 Inflammatory illness 11 Undifferentiated fever 12 Surgical abdomen 13 Other 14 Data not available |
| 255 | [out_disc_diagadd] | Additional information | text |
| 256 | [out_disc_sbi] | How would you categorise the final diagnosis based on Perform classification? | radio 1 Definite bacterial 2 Probable bacterial 3 Bacterial syndrome 4 Unknown bacterial or viral 5 Viral syndrome 6 Probable viral 7 Definite viral 8 Trivial 9 Other infection 10 Infection or inflammation 11 Inflammatory syndrome |
| 257 | [out_post_reattend] | Were there any unplanned repeat hospital attendances to the trust within 7 days of discharge? | radio 1 Yes 2 No 3 Data not available |
| 258 | [out_post_reattendreason] | Was the repeat hospital attendance(s) related to the same illness? *Show the field ONLY if: [out_post_reattend] = '1'* | radio 1 Yes 2 No 3 Data not available |
| 259 | [out_post_reattendelig] | Was the repeat hospital attendance(s) eligible for inclusion in FIRE? | radio 1 Yes 2 No 3 Data not available |
| 260 | [out_post_nextid] | What is the study ID of the subsequent hospital attendance(s)? *Show the field ONLY if: [out_post_reattendelig] = '1'* | text |
| 261 | [out_post_vital] | What was the vital status at 28 days from the date of hospital attendance? | radio 1 Alive 2 Dead 3 Data not available |
| 262 | [outcomes_complete] | Complete? | dropdown 0 Incomplete 1 Unverified 2 Complete |

# Supplementary Appendix 3 – NICE Guideline CG 143 adherence definitions

| Lower risk group | Infants aged **≥ 28 days** who appeared **well** to a healthcare professional AND had a **normal WCC** (5-15 x 10^9^/L). * |
| --- | --- |
| Higher risk group | Infants aged **< 28 days** OR  Infants aged **≥ 28 day**s who appeared **unwell** to a healthcare professional OR had an **abnormal WCC** (<5, >15 x 109/L) |

*Infants >=28 days and appearing well to healthcare professionals who did not have a FBC were included here.

|  | **Full adherence** | | **Non-adherence** | | **Partial adherence** | | **Over-adherence** | |
| --- | --- | --- | --- | --- | --- | --- | --- | --- |
|  | Lower risk group | Higher risk group | Lower risk group | Higher risk group | Lower risk group | Higher risk group | Lower risk group | Higher risk group |
| **CRP** | Yes | Yes | No | 0-3  Yes | 0-3  Yes | Yes | Yes | n/a |
| **WCC** | Yes | Yes | No |  |  | Yes | Yes |  |
| **Blood culture** | Yes | Yes | No |  |  | Yes | Yes |  |
| **Urine dip / MC&S** | Yes | Yes | No |  |  | Yes | Yes |  |
| **LP** | no | Yes | n/a | n/a | n/a | 0-1  Yes | 1-2  Yes |  |
| **Parenteral antibiotics** |  | Yes |  |  |  |  |  |  |

WCC – white cell count; FBC – full blood count; CRP - C-reactive protein; LP – lumbar puncture; MC&S – microscopy, culture & sensitivity

Full adherence:

Lower risk group who had a FBC and CRP and blood culture and a urine dip and/or urine MC&S

Higher risk group who had an FBC and CRP and blood culture, and a urine dip and/or MC&S, and LP and received parenteral antibiotics

Non-adherence:

Lower risk group who had none of: FBC, CRP, blood culture and urine dip and/or culture

Higher risk group who did not have the full set of the following four investigations: FBC, CRP, blood culture, urine dip and/or MC&S

Partial adherence:

Lower risk group who had one, two or three of the following four investigations: FBC, CRP, blood culture and urine dip and/or MC&S

Higher risk group who had a FBC and CRP and blood culture and urine dip and/or MC&S, but did not have an LP and/or did not receive parenteral antibiotics

Over-adherence:

Lower risk group who had an FBC and CRP and blood culture and urine dip and/or MC&S and had one or both of: LP and/or received parenteral antibiotics

WCC – white cell count; FBC – full blood count; CRP - C-reactive protein; LP – lumbar puncture; MC&S – microscopy, culture & sensitivity

# Supplementary Table S1 – Cohort characteristics: additional presenting features

|  | **Whole cohort** | **Age at presentation** | | | **Fever during assessment^1^** | | | **Time of presentation^2^** | | |
| --- | --- | --- | --- | --- | --- | --- | --- | --- | --- | --- |
|  |  | **<28 days** | **≥28 days** |  | **Febrile** | **Afebrile** |  | **Day** | **Night** |  |
|  | n=2,008 | n=450 | n=1558 | p | n=826 | n=1182 | p | n=1131 | n=877 | p |
| **Presenting features: Symptoms reported** | | | | | | | | | | |
| Reduced feeding | 774/2008  (38.5%) | 170/450  (37.8%) | 604/1558  (38.8%) | .704 | 348/826  (42.1%) | 426/1182  (36.0%) | **.006** | 446/1131  (39.4%) | 328/877  (37.4%) | .353 |
| Coryza | 750/2008 (37.4%) | 129/450  (28.7%) | 621/1558  (39.9%) | **<.001** | 294/826  (35.6%) | 456/1182 (38.6%) | .174 | 456/1131  (40.3%) | 294/877  (33.5%) | **.002** |
| Cough | 669/2008  (33.3%) | 92/450  (20.4%) | 557/1558  (37.0%) | **<.001** | 274/826 (33.2%) | 395/1182  (33.4%) | .908 | 400/1131  (35.4%) | 269/877  (30.7%) | **.027** |
| Lethargy | 372/2008 (18.5%) | 69/450  (15.3%) | 303/1558  (19.4%) | .**048** | 180/826  (21.8%) | 192/1182  (16.2%) | **.002** | 202/1131  (17.9%) | 170/877  (19.4%) | .383 |
| Irritability | 342/2008 (17.0%) | 82/450  (18.2%) | 260/1558  (16.7%) | .446 | 160/826 (19.4%) | 182/1182 (15.4%) | **.020** | 199/1131  (17.6%) | 143/877  (16.3%) | .446 |
| Vomiting | 337/2008 (16.8%) | 69/450  (15.3%) | 268/1558  (17.2%) | .350 | 117/826 (14.2%) | 220/1182 (18.6%) | .**009** | 192/1131  (17.0%) | 145/877  (16.5%) | .792 |
| Diarrhoea | 201/2008 (10.0%) | 33/450  (7.3%) | 168/1558  (10.8%) | **.032** | 72/826 (8.7%) | 129/1182 (10.9%) | .107 | 111/1131  (9.8%) | 90/877  (10.3%) | .740 |
| Reduced urine output | 114/2008  (5.7%) | 18/450  (4.0%) | 96/1558  (6.2%) | .081 | 43/826  (5.2%) | 71/1182 (6.0%) | .445 | 69/1131  (6.1%) | 45/877  (5.1%) | 352 |
| Reduced responsiveness to social cues | 21/2008  (1.0%) | 7/450  (1.6%) | 14/1558  (0.9%) | .289 | 16/826  (1.9%) | 5/1182 (0.4%) | **.001** | 11/1131  (1.0%) | 10/877  (1.1%) | .826 |
| Abdominal pain or distension | 20/2008  (1.0%) | 8/450  (1.8%) | 12/1558  (0.8%) | .100 | 8/826  (1.0%) | 12/1182  (1.0%) | .917 | 16/1131  (1.4%) | 4/877  (0.5%) | **.040** |
| Limb or joint swelling | 1/2007  (0.0%) | 1/450  (0.2%) | 0/1558  (0.0%) | .224 | 1/826  (0.1%) | 0/1182  (0.0%) | .411 | 1/1131  (0.1%) | 0/877  (0.0%) | .999 |
| None | 262/2008  (13.0%) | 68/450  (15.1%) | 194/1558  (12.5%) | .140 | 96/826  (11.6%) | 166/1182  (14.0%) | .113 | 127/1131  (11.2%) | 135/977  (15.4%) | **.006** |
| **Presenting features: Signs on examination** | | | | | | | | | | |
| Irritability | 132/2008  (6.6%) | 26/450  (5.8%) | 106/1558  (6.8%) | .439 | 78/826  (9.4%) | 54/1182  (4.6%) | <.001 | 70/1131  (6.2%) | 62/877  (7.1%) | .468 |
| Moderate or severe chest indrawing | 78/2008  (3.9%) | 17/450  (3.8%) | 61/1558  (3.9%) | .894 | 33/826  (4.0%) | 45/1182  (3.8%) | .907 | 50/1131  (4.4%) | 28/877  (3.2%) | .164 |
| Grunting | 47/2008  (2.3%) | 11/450  (2.4%) | 36/1558  (2.3%) | .869 | 31/826  (3.8%) | 16/1182  (1.4%) | **<.001** | 23/1131  (2.0%) | 24/877  (2.7%) | .303 |
| Lethargy | 42/2008  (2.1%) | 12/450  (2.7%) | 30/1558  (1.9%) | .333 | 24/826  (2.9%) | 18/1182  (1.5%) | **.039** | 30/1131  (2.7%) | 12/877  (1.4%) | .058 |
| Weak, high pitched or continuous cry | 25/2008  (1.2%) | 5/450  (1.1%) | 20/1558  (1.3%) | .999 | 15/826  (1.8%) | 10/1182  (0.8%) | .065 | 17/1131  (1.5%) | 8/877  (0.9%) | .311 |
| Abdominal distension | 17/2008  (0.8%) | 10/450  (2.2%) | 7/1558  (0.4%) | **.001** | 9/826  (1.1%) | 8/1182  (0.7%) | .333 | 10/1131  (0.9%) | 7/877  (0.8%) | .999 |
| Non-blanching rash/skin lesion > 2mm in diameter | 16/2008  (0.8%) | 2/450  (0.4%) | 14/1558  (0.9%) | .547 | 5/826  (0.6%) | 11/1182  (0.9%) | .459 | 10/1131  (0.9%) | 6/877  (0.7%) | .801 |
| Bulging fontanelle | 11/2008  (0.5%) | 2/450  (0.4%) | 9/1558  (0.6%) | .999 | 7/826  (0.8%) | 4/1182  (0.3%) | .217 | 5/1131  (0.4%) | 6/877  (0.7%) | .549 |
| No response to social cues | 7/2008  (0.3%) | 2/450  (0.4%) | 5/1558  (0.3%) | .657 | 6/826  (0.7%) | 1/1182  (0.1%) | **.022** | 4/1131  (0.4%) | 3/877  (0.3%) | .999 |
| Not rousable or if roused does not stay awake | 7/2008  (0.3%) | 2/450  (0.4%) | 5/1558  (0.3%) | .657 | 7/826  (0.8%) | 0/1182  (0.0%) | **.002** | 7/1131  (0.6%) | 0/877  (0.0%) | **.021** |
| Reduced urine output | 6/2008  (0.3%) | 2/450  (0.4%) | 4/1558  (0.3%) | .621 | 4/826  (0.5%) | 2/1182  (0.2%) | .236 | 5/1131  (0.4%) | 1/877  (0.1%) | .240 |
| Reduced skin turgor, skin doughy/tented, sunken eyes | 5/2008  (0.2%) | 3/450  (0.7%) | 2/1558  (0.1%) | .078 | 2/826  (0.2%) | 3/1182  (0.3%) | .999 | 2/1131  (0.2%) | 3/877  (0.3%) | .659 |
| No smile or face anxious, dull, expressionless | 5/2008  (0.2%) | 1/450  (0.2%) | 4/1558  (0.3%) | .999 | 3/826  (0.4%) | 2/1182  (0.2%) | .408 | 4/1131  (0.4%) | 1/877  (0.1%) | .394 |
| Swelling of a limb/joint | 2/2008  (0.1%) | 1/450  (0.2%) | 1/1558  (0.1%) | .398 | 2/826  (0.2%) | 0/1182  (0.0%) | .169 | 2/1131  (0.2%) | 0/877  (0.0%) | .508 |
| Neck stiffness | 1/2008  (0.0%) | 0/450  (0.0%) | 1/1558  (0.1%) | .999 | 1/826  (0.1%) | 0/1182  (0.0%) | .411 | 1/1131  (0.1%) | 0/877  (0.0%) | .999 |
| Seizure or focal neurological signs | 1/2008  (0.0%) | 1/450  (0.2%) | 0/1558  (0.0%) | .224 | 1/826  (0.1%) | 0/1182  (0.0%) | .411 | 0/1131  (0.0%) | 1/877  (0.1%) | .437 |
| None | 933/2008  (46.5%) | 217/350  (48.2%) | 716/1558  (46.0%) | .396 | 324/826  (39.2%) | 609/1182  (51.5%) | **<.001** | 514/1131  (45.4%) | 419/877  (47.8%) | .299 |
| **Presenting features: Vital signs** | | | | | | | | | | |
| Initial oxygen saturation <96% | 107/1932  (5.5%) | 25/433  (5.8%) | 82/1499  (5.5%) | .812 | 50/800  (6.3%) | 57/1132  (5.0%) | .267 | 66/1088  (6.1%) | 41/844  (4.9%) | .271 |

^1^ Fever during assessment was defined as recorded temperature ≥38°C at presentation or at any point whilst in the place of initial assessment (Emergency Department or acute assessment unit). Afebrile during assessment was defined as having a caregiver reported fever or feeling hot to touch in the 12 hours prior to presentation but temperature <38°C throughout stay in place of initial assessment

^2^ Day presentation was defined as presenting between 08:00-19:59 and night presentation as presenting between 20:00-07:59.

# Supplementary Table S2 – binary logistic regression analysis of variability in CSF sampling and administration of parenteral antibiotics

***S2a Binary logistic regression analysis of variability in CSF sampling***

| **Predictors** | | **Coefficient** | **Standard error** | **Odds ratio** | **Lower CI** | **Upper CI** | ***p* value** |
| --- | --- | --- | --- | --- | --- | --- | --- |
| Patient characteristics | Age (<28 days) | 0.90 | 0.13 | **2.46** | **1.89** | **3.20** | **<.001** |
|  | Sex (female) | -0.22 | 0.12 | 0.80 | 0.64 | 1.01 | .056 |
|  | Ethnicity (non-white) | -0.08 | 0.12 | 0.92 | 0.73 | 1.17 | .493 |
|  | IMDD (decile) |  |  |  |  |  | .285 |
|  | 1-2 | 0.21 | 0.24 | 1.23 | 0.77 | 1.96 | .381 |
|  | 3-4 | 0.05 | 0.22 | 1.05 | 0.69 | 1.61 | .805 |
|  | 5-6 | -0.02 | 0.22 | 0.98 | 0.64 | 1.50 | .931 |
|  | 7-8 | 0.33 | 0.21 | 1.40 | 0.92 | 2.13 | .120 |
|  | Birth gestation (term) | -0.48 | 0.24 | 0.62 | 0.38 | 1.00 | .050 |
|  | Comorbidities at high-risk of SBI | -0.09 | 0.40 | 0.92 | 0.42 | 1.99 | .823 |
| Presentation characteristics | Fever category (febrile) | 1.35 | 0.12 | **3.85** | **3.05** | **4.86** | **<.001** |
|  | Season of presentation |  |  |  |  |  | .245 |
|  | Winter | 0.25 | 0.16 | 1.29 | 0.95 | 1.75 | .109 |
|  | Spring | 0.33 | 0.17 | 1.39 | 0.99 | 1.95 | .057 |
|  | Summer | 0.18 | 0.16 | 1.20 | 0.87 | 1.65 | .273 |
|  | Day of presentation (weekend) | 0.13 | 0.13 | 1.14 | 0.88 | 1.46 | .320 |
|  | Time of presentation (daytime, %) | 0.16 | 0.12 | 1.18 | 0.94 | 1.48 | .155 |
| Site | Overall |  |  |  |  |  | **<.001** |
|  | B | -0.86 | 0.62 | 0.43 | 0.13 | 1.42 | .165 |
|  | C | 0.12 | 0.71 | 1.12 | 0.28 | 4.54 | .872 |
|  | D | -1.25 | 0.67 | 0.29 | 0.08 | 1.08 | .064 |
|  | E | 0.35 | 0.67 | 1.42 | 0.39 | 5.26 | .596 |
|  | F | -0.82 | 0.68 | 0.44 | 0.12 | 1.67 | .229 |
|  | G | -0.69 | 0.63 | 0.50 | 0.15 | 1.72 | .271 |
|  | H | -0.79 | 0.64 | 0.45 | 0.13 | 1.59 | .217 |
|  | I | -1.77 | 0.64 | **0.17** | **0.05** | **0.59** | **.005** |
|  | J | -0.65 | 0.65 | 0.52 | 0.15 | 1.85 | .312 |
|  | K | 0.71 | 0.65 | 2.03 | 0.57 | 7.25 | .274 |
|  | L | -0.60 | 0.63 | 0.55 | 0.16 | 1.90 | .346 |
|  | M | -0.01 | 0.66 | 0.99 | 0.27 | 3.58 | .988 |
|  | N | -1.15 | 0.67 | 0.32 | 0.09 | 1.18 | .087 |
|  | O | -0.54 | 0.62 | 0.58 | 0.17 | 1.98 | .389 |
|  | P | 0.02 | 0.63 | 1.02 | 0.30 | 3.48 | .980 |
|  | Q | -1.39 | 0.74 | 0.25 | 0.06 | 1.06 | .060 |
|  | R | 0.03 | 0.65 | 1.03 | 0.29 | 3.66 | .960 |
|  | S | -0.06 | 1.60 | 0.94 | 0.04 | 21.51 | .971 |
|  | T | 0.69 | 0.63 | 1.99 | 0.58 | 6.84 | .277 |
|  | Constant | -0.516 | 0.677 | 0.597 |  |  | .446 |

***S2b Binary logistic regression analysis of variability in the administration of parenteral antibiotics***

| **Predictors** | | **Coefficient** | **Standard error** | **Odds ratio** | **Lower CI** | | **Upper CI** | ***p* value** |
| --- | --- | --- | --- | --- | --- | --- | --- | --- |
| Patient characteristics | Age (<28 days) | 0.58 | 0.14 | **1.79** | **1.37** | **2.34** | | **<.001** |
|  | Sex (female) | -0.05 | 0.11 | 0.96 | 0.77 | 1.19 | | .687 |
|  | Ethnicity (non-white) | -0.16 | 0.12 | 0.86 | 0.68 | 1.08 | | .191 |
|  | IMDD (decile) |  |  |  |  |  | | .390 |
|  | 1-2 | -0.02 | 0.21 | 0.98 | 0.65 | 1.47 | | .918 |
|  | 3-4 | -0.30 | 0.21 | 0.74 | 0.49 | 1.11 | | .149 |
|  | 5-6 | -0.21 | 0.21 | 0.81 | 0.54 | 1.22 | | .315 |
|  | 7-8 | -0.04 | 0.23 | 0.97 | 0.61 | 1.53 | | .880 |
|  | Birth gestation (term) | -0.53 | 0.25 | **0.59** | **0.36** | **0.97** | | **.036** |
|  | Comorbidities at high-risk of SBI | 0.31 | 0.42 | 1.36 | 0.59 | 3.11 | | .466 |
| Presentation characteristics | Fever category (febrile) | 1.30 | 0.12 | **3.65** | **2.89** | **4.62** | | **<.001** |
|  | Season of presentation |  |  |  |  |  | | .226 |
|  | Winter | 0.19 | 0.15 | 1.21 | 0.90 | 1.63 | | .210 |
|  | Spring | 0.16 | 0.17 | 1.18 | 0.85 | 1.64 | | .332 |
|  | Summer | 0.33 | 0.16 | **1.39** | **1.02** | **1.90** | | **.038** |
|  | Day of presentation (weekend) | 0.15 | 0.13 | 1.16 | 0.90 | 1.49 | | .251 |
|  | Time of presentation (daytime, %) | 0.11 | 0.11 | 1.11 | 0.89 | 1.39 | | .345 |
| Site | Overall |  |  |  |  |  | | **<.001** |
|  | B | -0.88 | 0.65 | 0.41 | 0.12 | 1.49 | | .176 |
|  | C | -0.17 | 0.76 | 0.84 | 0.19 | 3.71 | | .822 |
|  | D | -1.33 | 0.69 | 0.26 | 0.07 | 1.02 | | .054 |
|  | E | 0.29 | 0.71 | 1.34 | 0.33 | 5.43 | | .680 |
|  | F | -0.10 | 0.73 | 0.90 | 0.22 | 3.74 | | .886 |
|  | G | -0.51 | 0.66 | 0.60 | 0.16 | 2.20 | | .441 |
|  | H | -0.65 | 0.67 | 0.52 | 0.14 | 1.95 | | .332 |
|  | I | -1.45 | 0.66 | 0.24 | **0.06** | **0.86** | | **.029** |
|  | J | -0.59 | 0.68 | 0.55 | 0.15 | 2.09 | | .382 |
|  | K | 0.96 | 0.71 | 2.61 | 0.65 | 10.49 | | .176 |
|  | L | -0.83 | 0.67 | 0.44 | 0.12 | 1.62 | | .215 |
|  | M | 0.49 | 0.72 | 1.63 | 0.40 | 6.67 | | .495 |
|  | N | -0.99 | 0.69 | 0.37 | 0.10 | 1.44 | | .151 |
|  | O | -0.84 | 0.66 | 0.43 | 0.12 | 1.57 | | .202 |
|  | P | -0.12 | 0.67 | 0.88 | 0.24 | 3.29 | | .855 |
|  | Q | -0.63 | 0.74 | 0.53 | 0.12 | 2.25 | | .390 |
|  | R | -0.23 | 0.68 | 0.80 | 0.21 | 3.05 | | .740 |
|  | S | -0.93 | 1.69 | 0.40 | 0.01 | 10.81 | | .583 |
|  | T | 1.29 | 0.71 | 3.64 | 0.91 | 14.57 | | .067 |
|  | Constant | 0.659 | 0.714 | 1.933 |  |  | | .356 |

All variables are categoric. **Patient characteristics:** Age <28 days (reference category: ≥28 days), sex female (ref: male), ethnicity non-white (ref: any white ethnicity), IMDD (ref:deciles 9-10), birth gestation term (ref: preterm 34-37 weeks), co-morbidities at high risk of SBI (as previously defined, ref: no comorbidities or comorbidities not at high risk for SBI). **Presentation characteristics:** Fever category febrile (categories as previously described, ref: afebrile), day of presentation weekend (ref: any weekday), season of presentation (ref: autumn), time of presentation daytime (ref: nighttime). **Sites:** anonymised hospital sites (ref: hospital site A)

The use of an appropriate thermometer at home or administration of antipyretics prior to presentation were not included as variables in the model due to missing data.

CSF sampling: R2 (Cox and Snell) = 0.193

Administration of parenteral antibiotics: R2 (Cox and Snell) = 0.178

# Supplementary Table S3 – Comparison of investigations and management between subgroups

| **Age at presentation** | | | | | | |
| --- | --- | --- | --- | --- | --- | --- |
|  | Age <28 days | | Age >=28 days | | P value | Odds ratio  (95% confidence interval) |
| FBC | 362/450 | 80.4% | 1117/1558 | 71.7% | <0.001 | 1.62 (1.26-2.10) |
| CRP | 364/450 | 80.9% | 1124/1558 | 72.1% | <0.001 | 1.63 (1.26-2.10) |
| Blood culture | 324/450 | 72.0% | 928/1558 | 59.6% | <0.001 | 1.75 (1.39-2.20) |
| Urine | 312/450 | 69.3% | 961/1558 | 61.7% | 0.003 | 1.41 (1.12-1.76) |
| CSF | 253/450 | 56.2% | 566/1558 | 36.3% | <0.001 | 2.25 (1.82-2.79) |
| CXR | 63/450 | 14.0% | 170/1558 | 10.9% | 0.072 | 1.33 (0.98-1.81) |
| Parenteral antibiotics* | 305/450 | 67.8% | 853/1558 | 54.7% | <0.001 | 1.24 (1.15-1.34) |
| Inpatient admission | 335/450 | 74.4% | 924/1558 | 59.3% | <0.001 | 1.26 (1.17-1.34) |
| **Febrile during assessment** | | | | | | |
|  | Febrile | | Afebrile | | P value | Odds ratio  (95% confidence interval) |
| FBC | 717/826 | 86.8% | 762/1182 | 64.5% | <0.001 | 3.63 (2.87-4.58) |
| CRP | 718/826 | 86.9% | 770/1182 | 65.1% | <0.001 | 3.56 (2.81-4.50) |
| Blood culture | 650/826 | 78.7% | 602/1182 | 50.9% | <0.001 | 3.56 (2.91-4.35) |
| Urine | 616/826 | 74.6% | 657/1182 | 55.6% | <0.001 | 2.34 (1.93-2.85) |
| CSF | 473/826 | 57.3% | 346/1182 | 29.3% | <0.001 | 3.24 (2.69-3.90) |
| CXR | 124/826 | 15.0% | 109/1182 | 9.2% | <0.001 | 1.74 (1.32-2.29) |
| Parenteral antibiotics* | 609/826 | 73.7% | 549/1182 | 46.4% | <0.001 | 1.59 (1.48-1.71) |
| Inpatient admission | 648/826 | 78.5% | 611/1182 | 51.7% | <0.001 | 1.52 (1.42-1.62) |
| **Time of day at presentation** | | | | | | |
|  | Day presentation | | Night presentation | | P value | Odds ratio  (95% confidence interval) |
| FBC | 846/1131 | 74.8% | 633/877 | 72.2% | 0.186 | 1.14 (0.94-1.40) |
| CRP | 852/1131 | 75.3% | 636/877 | 72.5% | 0.154 | 1.16 (0.95-1.41) |
| Blood culture | 702/1131 | 62.1% | 550/877 | 62.7% | 0.767 | 0.97 (0.81-1.17) |
| Urine | 727/1131 | 64.3% | 546/877 | 62.3% | 0.351 | 1.09 (0.91-1.31) |
| CSF | 452/1131 | 55.2% | 367/877 | 41.8% | 0.395 | 0.93 (0.77-1.11) |
| CXR | 126/1131 | 11.1% | 107/877 | 12.2% | 0.462 | 0.90 (0.69-1.19) |
| Parenteral antibiotics* | 641/1131 | 56.7% | 517/877 | 59.0% | 0.306 | 0.96 (0.89-1.04) |
| Inpatient admission | 701/1131 | 62.0% | 558/319 | 63.6% | 0.45 | 0.97 (0.91-1.04) |

* parental antibiotics: intravenous or intramuscular administration, given in ward inpatient or ambulating setting.

FBC – full blood count; CRP - C-reactive protein; CSF– cerebrospinal fluid (sampled by lumbar puncture); CXR – chest x-ray

# Supplementary Table S4 – Adherence to national clinical practice guideline BSAC “Guideline for infants under 3 months with a fever and no source” in relation to investigation and management of febrile infants

| **Guideline domain** | **Guideline standard** | **Whole cohort** | **Febrile during assessment** | **Afebrile during assessment** |
| --- | --- | --- | --- | --- |
|  |  | *actual/expected (%)* | *actual/expected (%)* | *actual/expected (%)* |
| Lumbar puncture | <1 month | 253/450 (56.2%) | 146/182 (80.2%) | 107/268 (39.9%) |
|  | Clinical meningitis | 92/122 (75.4%) | 59/74 (79.7%) | 33/48 (68.8%) |
|  | Appear unwell, aged >1 month | 46/65 (70.8%) | 32/41 (78.0%) | 14/24 (58.3%) |
|  | Comorbidities high risk for SBI ^a^ | 17/41 (41.5%) | 8/16 (50.0%) | 9/25 (36.0%) |
|  | Any indication above | 384/650 (59.1%) | 227/292 (77.7%) | 157/358 (43.9%) |
| No IV antibiotics | Low risk ^b^ | 610/1077 (56.6%) | 174/408 (42.6%) | 436/669 (65.2%) |
| IV antibiotics | Moderate risk ^c^ | 293/356 (82.3%) | 154/174 (88.5%) | 139/182 (76.4%) |
|  | High risk ^d^ | 398/575 (69.2%) | 221/244 (90.6%) | 177/331 (53.5%) |
| Colour coding: green ≥80%, orange 60-80%, red <60%. | | | | |
| British Society for Antimicrobial Chemotherapy (BSAC): Guideline for infants under 3 months with a fever and no source | | | | |
| ^a^ “Comorbidities high risk for serious bacterial infection (immunosuppression, indwelling devices etc)” as listed on BSAC guidance [4] was taken to include also significant cardiac or gastrointestinal comorbidites | | | | |
| ^b^ Low risk: Age >28 days and inflammatory markers not raised (CRP <20mg/L and neutrophil count <10x10^9^/L) and not unwell appearing and negative urinalysis | | | | |
| ^c^ Moderate risk: Age >28 days and not unwell appearing and either inflammatory markers raised  (CRP ≥20mg/L or neutrophil count ≥10x10^9^/L) and/or positive urinalysis | | | | |
| ^d^ High risk: Age <28 days or unwell appearing or comorbidities high risk for serious bacterial infection (included infants with significant cardiac or gastrointestinal comorbidities and those with abnormalities of the renal tract requiring antibiotic prophylaxis) | | | | |

# Supplementary Table S5 – NICE Guideline CG 143 adherence by age and fever during assessment

|  | **Full adherence** | | **Partial adherence** | | **Non-adherence** | | **Over-adherence** | |
| --- | --- | --- | --- | --- | --- | --- | --- | --- |
| Whole cohort | 440/2008 | 21.9% | 490/2008 | 24.4% | 627/2008 | 31.2% | 451/2008 | 22.5% |
| Whole cohort - fever measured in hospital | 246/826 | 29.8% | 185/826 | 22.4% | 156/826 | 18.9% | 239/826 | 28.9% |
| Whole cohort - fever reported at home only | 194/1182 | 16.4% | 305/1882 | 25.8% | 471/1182 | 39.8% | 212/1182 | 17.9% |
|  |  |  |  |  |  |  |  |  |
| <28 days of age | 213/450 | 47.3% | 59/450 | 13.1% | 178/450 | 39.6% | n/a | n/a |
| <28 days of age - fever measured in hospital | 125/182 | 68.7% | 23/182 | 12.6% | 34/182 | 18.7% | n/a | n/a |
| <28 days of age - fever reported at home only | 88/268 | 32.8% | 36/268 | 13.4% | 144/268 | 53.7% | n/a | n/a |
|  |  |  |  |  |  |  |  |  |
| ≥28 days of age | 227/1558 | 14.6% | 431/1558 | 27.7% | 449/1558 | 28.8% | 451/1558 | 28.9% |
| ≥28 days of age - fever measured in hospital | 121/644 | 18.8% | 162/644 | 25.2% | 122/644 | 18.9% | 239/644 | 37.1% |
| ≥28 days of age - fever reported at home only | 106/914 | 11.6% | 269/914 | 29.4% | 327/914 | 35.8% | 212/914 | 23.2% |
|  |  |  |  |  |  |  |  |  |
| Afebrile during assessment |  |  |  |  |  |  |  |  |
| With appropriate* thermometer used at home** | 64/292 | 21.9% | 83/292 | 28.4% | 73/292 | 25.0% | 72/292 | 24.7% |
| With inappropriate thermometer used at home | 31/299 | 10.4% | 160/299 | 53.5% | 77/299 | 25.8% | 31/299 | 10.4% |
| With antipyretics*** given at home**** | 34/297 | 11.4% | 119/297 | 40.1% | 82/297 | 27.6% | 62/297 | 20.9% |
| With no antipyretics given at home | 99/529 | 18.7% | 198/529 | 37.4% | 134/529 | 25.3% | 98/529 | 18.5% |
|  |  |  |  |  |  |  |  |  |
| * Appropriate thermometer defined as use of axillary thermometer in infants aged <28 days and use of axillary and/or tympanic thermometer in infants aged ≥28 days of age | | | | | | | | |
| ** Data not available for method of temperature assessment at home = 591/1182 (50.0%) | | | |  |  |  |  |  |
| *** Any antipyretic given in the 8 hours prior to presentation | |  |  |  |  |  |  |  |
| **** Data not available for administration of antipyretics at home = 356/1182 (30.1%) | | | |  |  |  |  |  |

# Supplementary Table S6 – NICE Guideline CG 143 adherence by risk group

|  | **Lower risk group** | | | **Higher risk group** | | |
| --- | --- | --- | --- | --- | --- | --- |
| **Full adherence** | **37/1175** | **(3.1%)** | | **403/833** | **(48.4%)** | |
| **Non-adherence** | **340/1175** | **(28.9%)** | | **287/833** | **(34.5%)** | |
|  | n/a  (all had no investigations nor parenteral antibiotics) | | | Missing FBC | 89/287 | (31.0%) |
|  |  |  |  | Missing CRP | 88/287 | (30.7%) |
|  |  |  |  | Missing blood culture | 181/287 | (63.1%) |
|  |  |  |  | Missing urine dip and/or MC&S | 226/287 | (78.7%) |
|  |  |  |  |  |  |  |
|  |  |  |  | 1/4 investigations missing | 146/287 | (50.9%) |
|  |  |  |  | 2/4 investigations missing | 53/287 | (18.5%) |
|  |  |  |  | 3/4 investigations missing | 20/287 | (7.0%) |
|  |  |  |  | 4/4 investigations missing | 68/287 | (23.7%) |
| **Partial adherence** | **347/1175** | **(29.5%)** | | **143/833** | **(17.2%)** | |
|  | Missing FBC | 100/347 | (28.8%) | Missing LP only | 108/143 | (75.5%) |
|  | Missing CRP | 92/347 | (26.5%) | Missing parenteral antibiotics only | 13/143 | (9.1%) |
|  | Missing blood culture | 235/347 | (67.7%) | Missing LP and parenteral antibiotics | 22/142 | (15.4%) |
|  | Missing urine dip and/or MC&S | 169/347 | (48.7%) |  |  |  |
|  |  |  |  |  |  |  |
|  | 1/4 investigations missing | 191/347 | (55.0%) |  |  |  |
|  | 2/4 investigations missing | 63/347 | (18.2%) |  |  |  |
|  | 3/4 investigations missing | 93/347 | (36.8%) |  |  |  |
| **Over-adherence** | **451/1175** | **(38.4%)** | | **n/a** |  |  |
|  | Additional LP only | 7/451 | (1.6%) |  |  |  |
|  | Additional parenteral antibiotics only | 151/451 | (33.5%) |  |  |  |
|  | Additional LP and parenteral antibiotics | 293/451 | (65.0%) |  |  |  |

* parental antibiotics: intravenous or intramuscular administration, given in ward inpatient or ambulating setting.

FBC – full blood count; CRP - C-reactive protein; MC&S – microscopy, culture & sensitivity; LP lumbar puncture; CXR – chest x-ray

# Supplementary Table S7 – Binary logistic regression analysis of predictors for adherence (appropriate vs. inappropriate management) to NICE guideline CG 143

| **Predictors** | | **Coefficient** | **Standard error** | **Odds ratio** | **Lower CI** | **Upper CI** | ***p* value** |
| --- | --- | --- | --- | --- | --- | --- | --- |
| **Patient characteristics** | Age (<28 days) | 1.765 | 0.139 | **5.840** | **4.448** | **7.667** | **<.001** |
|  | Sex (female) | -0.314 | 0.136 | **0.730** | **0.559** | **0.954** | **.021** |
|  | Ethnicity (non-white) | -0.017 | 0.137 | 0.983 | 0.752 | 1.286 | .903 |
|  | IMDD (decile) |  |  |  |  |  | .231 |
|  | 1-2 | 0.231 | 0.243 | 1.260 | 0.782 | 2.028 | .342 |
|  | 3-4 | -0.206 | 0.224 | 0.814 | 0.525 | 1.262 | .358 |
|  | 5-6 | -0.108 | 0.240 | 0.898 | 0.561 | 1.438 | .654 |
|  | 7-8 | -0.068 | 0.246 | 0.934 | 0.577 | 1.512 | .782 |
|  | Birth gestation (term) | -0.044 | 0.298 | 0.957 | 0.534 | 1.716 | .882 |
|  | Comorbidities at high-risk of SBI | 0.148 | 0.458 | 1.159 | 0.472 | 2.844 | .747 |
| **Presentation characteristics** | Fever category (febrile) | 0.913 | 0.134 | **2.493** | **1.917** | **3.242** | **<.001** |
|  | Season of presentation |  |  |  |  |  | .688 |
|  | Winter | 0.083 | 0.183 | 1.086 | 0.759 | 1.555 | .651 |
|  | Spring | 0.073 | 0.200 | 1.076 | 0.727 | 1.593 | .714 |
|  | Summer | 0.217 | 0.185 | 1.243 | 0.865 | 1.786 | .240 |
|  | Day of presentation (weekend) | 0.010 | 0.150 | 1.010 | 0.753 | 1.354 | .948 |
|  | Time of presentation (daytime) | -0.024 | 0.133 | 0.976 | 0.752 | 1.267 | .855 |
| **Site characteristics** | Type of hospital (DGH) | 0.245 | 0.162 | 1.278 | 0.931 | 1.754 | .129 |
|  | Guideline usage (local CPG) | 0.025 | 0.135 | 1.025 | 0.786 | 1.336 | .856 |
|  | Constant | -2.449 | 0.428 | 0.086 |  |  | **<.001** |

All variables are categoric. **Patient characteristics:** Age <28 days (reference category: ≥28 days), sex female (ref: male), ethnicity non-white (ref: any white ethnicity), IMDD (ref:deciles 9-10), birth gestation term (ref: preterm 34-37 weeks), co-morbidities at high risk of SBI (as previously defined, ref: no comorbidities or comorbidities not at high risk for SBI). **Presentation characteristics:** Fever category febrile (categories as previously described, ref: afebrile), day of presentation weekend (ref: any weekday), season of presentation (ref: autumn), time of presentation daytime (ref: nighttime). **Site characteristics:** type of hospital DGH (ref:. tertiary), guideline usage local CPG in use (ref: hospital site without local CPG).

The use of an appropriate thermometer at home or administration of antipyretics prior to presentation were not included as variables in the model due to missing data.

R2 (Cox and Snell) = 0.131
